# The Roles of Histone Deacetylases and Their Inhibitors in Cancer Therapy

**DOI:** 10.3389/fcell.2020.576946

**Published:** 2020-09-29

**Authors:** Guo Li, Yuan Tian, Wei-Guo Zhu

**Affiliations:** ^1^Guangdong Key Laboratory for Genome Stability and Human Disease Prevention, Department of Biochemistry and Molecular Biology, School of Basic Medical Sciences, Shenzhen University Health Science Center, Shenzhen, China; ^2^Shenzhen Bay Laboratory, Shenzhen, China

**Keywords:** cancer therapy, HDAC, HDAC inhibitors, HDAC sequence, histone modification

## Abstract

Genetic mutations and abnormal gene regulation are key mechanisms underlying tumorigenesis. Nucleosomes, which consist of DNA wrapped around histone cores, represent the basic units of chromatin. The fifth amino group (N^ε^) of histone lysine residues is a common site for post-translational modifications (PTMs), and of these, acetylation is the second most common. Histone acetylation is modulated by histone acetyltransferases (HATs) and histone deacetylases (HDACs), and is involved in the regulation of gene expression. Over the past two decades, numerous studies characterizing HDACs and HDAC inhibitors (HDACi) have provided novel and exciting insights concerning their underlying biological mechanisms and potential anti-cancer treatments. In this review, we detail the diverse structures of HDACs and their underlying biological functions, including transcriptional regulation, metabolism, angiogenesis, DNA damage response, cell cycle, apoptosis, protein degradation, immunity and other several physiological processes. We also highlight potential avenues to use HDACi as novel, precision cancer treatments.

## Introduction

In the nuclei of eukaryotic cells, the entire genome of an organism is condensed into chromatin. The nucleosome is the basic unit of chromatin: it contains 147 DNA base pairs coiled around a core histone octamer, which includes histones H2A, H2B, H3 and H4 (Luger et al., 1997; Tessarz and Kouzarides, 2014). The additional linker histone H1 interacts with chromatin outside of the core octamer to regulate higher order chromatin structure (Fyodorov et al., 2018). There are two major higher order structures: heterochromatin refers to condensed chromatin, and euchromatin refers to loosely packed chromatin that is more accessible to transcriptional regulators and RNA polymerase complexes (Allfrey et al., 1964). Thus, alteration and regulation of chromatin structure impacts gene expression by making certain genes more or less available for transcription.

The epigenome is comprised of modifications to chromatin, including DNA methylation and histone modifications. For example, DNA accessibility is regulated by nucleosome sliding or post-translational modifications (PTMs), which include phosphorylation, methylation and acetylation. These covalent modifications control the structure and function of chromatin through a number of regulators. These regulators can be broadly divided into “readers” (enzymes that bind to modifications and facilitate epigenetic activities), “writers”(enzymes that establish DNA methylation or histone modifications), and “erasers”(enzymes that remove these markers) (Taverna et al., 2007; Kutateladze, 2011; Musselman et al., 2012; Huang et al., 2014; Xu et al., 2017). As an example, acetylation occurs at the fifth NH2 (N^ε^) of histone lysine residues (Strahl and Allis, 2000; Jenuwein and Allis, 2001), and is read by the bromodomain-containing protein (BRD), written by histone acetyltransferases (HATs), and erased by histone deacetylases (HDACs) (Dawson and Kouzarides, 2012; Filippakopoulos and Knapp, 2014; Zaware and Zhou, 2019).

The acetylation of lysine residues (Kac) on histone tails generates positive charges, which neutralize negatively charged DNA and the unwinding of tightly coiled heterochromatin (Shogren-Knaak et al., 2006). Histone acetylation can increase the inner pore space of chromatin from 20 nm to 60−100 nm, altering spatial distance and accessibility during interphase (Gorisch et al., 2005); it also ensures sufficient space for local transcriptional events, including initiation and elongation (Wang et al., 2009). Acetylation is of particular importance because the interaction between histones and chromatin is generally very stable, and interruption of this interaction requires a high concentration of NaCl or acetate (Von Holt et al., 1989; Shechter et al., 2007). Notably, acetylation is often a necessary precursor to other modifications, such as phosphorylation, methylation and ubiquitylation (Yang and Gregoire, 2007; Yang and Seto, 2008a).

Acetylation is controlled by two antagonistic enzyme families: HATs and HDACs. HDACs are expressed by various tumors, and are involved in vital chromosomal translocation-mediated oncogenic protein fusion and carcinogenic events (Falkenberg and Johnstone, 2014; West and Johnstone, 2014). These enzymes were first revealed to remove acetyl groups from histones by Vincent Allfrey (Inoue and Fujimoto, 1969). The first HDAC that was discovered, HDAC1, was originally isolated by utilizing a microbe-derived cyclic tetrapeptide, Trapoxin, which inhibits histone deacetylation and induces cell-cycle arrest (Taunton et al., 1996). Sequence homology-dependent HDACs were subsequently identified, and shown to be involved in major biological functions such as transcription, metastasis, autophagy, cell cycle, DNA damage repair, angiogenesis, stress responses and senescence (Yang and Seto, 2008b; Li and Zhu, 2014).

Histone deacetylase inhibitors (HDACi) might be able to reverse the activation of tumor suppressor genes (TSG), and in this way inhibit the viability and malignant proliferation of tumor cells (Glozak and Seto, 2007). The efficacy of HDACi treatment has been demonstrated in numerous clinical studies. This review discusses HDACs and their inhibitors in the context of potential cancer treatments.

## Classifications, Enzymatic Activities and Cellular Distributions of HDACs

### Classifications of HDACs

**FIGURE 1:**
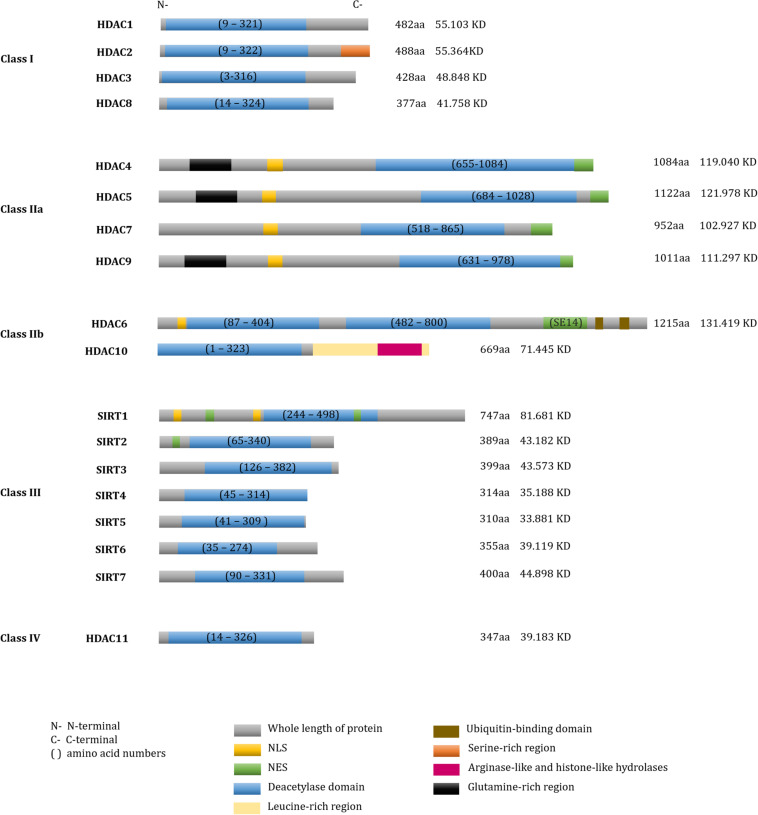
Domain structure of human HDACs. The fundamental structure of all deacetylases. Total number of amino acid residues and molecular weights in each HDAC were shown on the right of each protein.

According to their sequence similarities with yeast HDACs, 18 human HDACs have been identified and grouped into four classes (Yang and Seto, 2008b; Seto and Yoshida, 2014). Class I HDACs include HDAC1, -2, -3, and -8 (Rundlett et al., 1996; Taunton et al., 1996; Yang et al., 1996, 1997; Emiliani et al., 1998; Buggy et al., 2000; Hu et al., 2000; Van Den Wyngaert et al., 2000). Class II HDACs are further divided into two subgroups: class IIa and class IIb. Class IIa includes HDAC4, -5, -7, and -9 and class IIb includes HDAC6 and -10 (Grozinger et al., 1999; Miska et al., 1999; Wang et al., 1999; Kao et al., 2000, 2002; Zhou et al., 2001; Fischer et al., 2002; Guardiola and Yao, 2002; Tong et al., 2002). Class III, also known as the sirtuins (SIRTs), include SIRT1-7 (Imai et al., 2000; Lin et al., 2000), and class IV contains only HDAC11 (Gao et al., 2002). SIRTs are nicotinamide adenine dinucleotide (NAD^+^)-dependent enzymes, while the other three classes are Zinc cation (or Zn^2+^ ion)-dependent HDACs. Besides the deacetylase activity, a number of diverse enzymatic activities of HDACs are presented in Table 1 and the sequence characteristics of HDACs are presented in Figure 1.

**TABLE 1:** The multifaceted catalytic functions of HDACs.

Enzymatic activities	HDACs	References
deacetylase	All HDACs	−
polyamine deacetylase	HDAC10	Hai et al., 2017
fatty acid deacylase (de-hexanoyl, de-octanoyl, de-octanoyl, de-dodecanoyl, de-myristoyl)	HDAC8, HDAC11, SIRT6	Houtkooper et al., 2012; Feldman et al., 2013; Jiang et al., 2013; Aramsangtienchai et al., 2016; Wang et al., 2016; Kutil et al., 2018
decrotonylase	HDAC1, HDAC3	Wei et al., 2017
desuccinylase	SIRT5, SIRT7	Du et al., 2011; Li et al., 2016
demalonylase	SIRT5	Du et al., 2011
deglutarylase	SIRT5, SIRT7	Tan et al., 2014; Bao et al., 2019
de-methylglutarylase	SIRT4	Anderson et al., 2017
de-hydroxymethylglutarylase	SIRT4	Anderson et al., 2017
de-3-methylglutaconylase	SIRT4	Anderson et al., 2017
lipoamidase	SIRT4	Mathias et al., 2014
ADP-ribosyltransferase	SIRTs	Tanny et al., 1999; Haigis et al., 2006; Mao et al., 2011
S-nitrosylase	HDAC2	Nott et al., 2008; Cencioni et al., 2018
SUMOylase	HDAC4, HDAC7	Zhao et al., 2005; Gao et al., 2008; Yang Y. et al., 2011
O-GlcNAcylation	HDAC1, HDAC4, HDAC6, SIRT1	Zhu et al., 2016; Ferrer et al., 2017; Kronlage et al., 2019; Tian and Qin, 2019
S-glutathionylase	SIRT1	Brautigam et al., 2013
benzoylase	SIRT2	Huang et al., 2018a

### Compositions, Sequence Characteristics, and Cellular Distributions of HDACs

#### Class I HDACs

Of class I HDACs, HDAC1, -2, and -3 catalytic activities depend on their respective co-repressor complexes. Based on the conserved structures and dimerization domains, HDAC1 and HDAC2 are often recruited to the same co-repressor complexes, including Mi-2/nucleosome remodeling deacetylase (NuRD), repressor element-1 silencing transcription co-repressor (RCOR1/CoREST), SWI-independent-3A (Sin3A) and mitotic deacetylase complex (MiDAC) (Hassig et al., 1997; Laherty et al., 1997; Nagy et al., 1997; Ayer, 1999; You et al., 2001; Bantscheff et al., 2011; Itoh et al., 2015; Turnbull et al., 2020). HDAC3 associates with the nuclear receptor co-repressor (NCoR) and silencing mediator for retinoid or thyroid-hormone receptors (SMRT) to form co-repressors. The NCoR/SMRT complex provides a platform for the recruitment and activation of HDAC3 in the deacetylase-activating domain (DAD) of SMRT (Wen et al., 2000; Oberoi et al., 2011; Emmett and Lazar, 2019). Inositol phosphate, an intermolecular “glue”; binds to the interface between the co-repressors and HDAC catalytic domains, improving the catalytic activity of the HDACs in NuRD and NCoR/SMRT complexes (Watson et al., 2012, 2016; Millard et al., 2013). Particularly, the HDAC8 monomer accommodates substrates with a unique flexible L1 loop in its N-terminal region, which is absent in other HDACs (Somoza et al., 2004). Therefore, this motif is likely to be conducive to the development of HDAC8-specific inhibitors (Ingham et al., 2016). Furthermore, its crystal structure indicates that dimerization occurs at the binding interface between HDAC8 and its substrate (Castaneda et al., 2017).

#### Class II HDACs

All Class IIa HDACs include an extended N-terminal domain that contains conserved serine (Ser) residues and other motifs for localization and function (Yang and Gregoire, 2005). Based on these Ser residues, several kinases such as calcium/calmodulin-dependent protein kinase (CaMK), salt-inducible kinase (SIK) and members of microtubule affinity-regulating kinase (MARK/hPar-1) phosphorylate Class IIa HDACs (Mckinsey et al., 2000, 2001; Dequiedt et al., 2006; Walkinshaw et al., 2013), which facilitates HDACs nuclear export through chromosomal region maintenance 1 protein (CRM1) [also called exportin 1 (XPO1)]- or ankyrin repeat family A protein 2 (ANKRA2)-recognized nuclear export sequence (NES) (Wang and Yang, 2001; Mckinsey et al., 2006; Xu et al., 2012). For nuclear localization, all class IIa HDACs contain nuclear localization sequence (NLS) (Zhang et al., 2002b). 14-3-3 protein inhibits the nuclear localization of these HDACs by blocking their interaction with importin α. The absence of 14-3-3 promotes HDAC4/5 nuclear localization, which also facilitates transcription repression by binding to HDAC3 (Grozinger and Schreiber, 2000; Wang et al., 2000). Of note, Class IIa HDACs contains myocyte enhancer factor 2 (MEF2) binding sites. The phosphorylated kinases-induced exported HDACs dissociate with nuclear MEF2 family proteins that are response for differentiated gene expression (Sparrow et al., 1999; Wang et al., 1999; Mckinsey et al., 2000; Walkinshaw et al., 2013). Nevertheless, Class IIa HDACs have significant weaker deacetylase activity compared to Class I. X-ray crystallography data have revealed that the catalytic pocket of histone deacetylase-like protein (HDLP) contains several key catalytic sites, such as histidine (His), aspartic acid (Asp) and tyrosine (Tyr). Class IIa HDACs have relatively low catalytic ability due to a substitution of asparagine to Asp on the Asp-His charge relay (Finnin et al., 1999). Moreover, the catalytic Tyr is conserved in other HDACs except for class IIa enzymes, where the Tyr residue is replaced by His. Substitution of His back to Tyr at 976 recovers the enzymatic activity of class IIa HDACs (Lahm et al., 2007).

Of the class IIb HDACs, HDAC6 is a microtubule-associated deacetylase that is predominantly localized in the cytoplasm. HDAC6 contains a microtubule-binding domain that promotes chemotactic cell motility (Hubbert et al., 2002; Ustinova et al., 2020); it also includes a double-tandem deacetylase domain and a serine-glutamine containing tetradecapeptide (SE14) repeats domain that is important for cytoplasmic anchoring (Bertos et al., 2004). HDAC6 undergoes nuclear export via leucine-rich motifs that are recognized by CRM1/exportin1 (Verdel et al., 2000), and contains NLS at adjacent Kac sites in the N-terminal (Liu et al., 2012). HDAC6 also contains zinc-finger ubiquitin binding domains (ZnF-UBP, also called PAZ domain) that negatively regulate polyubiquitin chain turnover (Seigneurin-Berny et al., 2001; Hook et al., 2002; Boyault et al., 2006). Recently, two deacetylase domains of HDAC6 have been re-classified as catalytic domain 1 (CD1) and CD2: these domains confer differential substrate recognition (Hai and Christianson, 2016).

HDAC10 is a polyamine deacetylase that preferentially catalyzes N^8^-acetylspermidine hydrolysis to generate acetate (Hai et al., 2017; Shinsky and Christianson, 2018). It contains a leucine-rich domain, a deacetylase domain and an inactivity domain (Guardiola and Yao, 2002; Kao et al., 2002). Similar to class IIa HDACs, HDAC10 associates with HDAC2, HDAC3, SMRT, and NCOR2 to enhance transcriptional repression (Fischer et al., 2002; Tong et al., 2002; Jin et al., 2018).

#### Class III HDACs

A total of seven NAD^+^-dependent class III HDACs, or SIRTs, have been identified in the cytoplasm, nucleus and mitochondria (Yao et al., 2014; Chalkiadaki and Guarente, 2015; Zhao and Zhou, 2019). SIRT1, which is distributed in the cytoplasm, mitochondria and the nucleus, has two CRM1-mediated NES and two NLS (Tanno et al., 2007): it undergoes conformational shifts in response to adenosine triphosphate (ATP), which impedes its ability to interact with substrates in the C-terminal domain (Kang et al., 2017). SIRT2 is even more widely distributed than SIRT1, being found in the plasma membrane and cytoskeleton-associated organelles in addition to the cytoplasm, nucleus and mitochondria: it contains a CRM1-dependent NES and a putative leucine-rich NES (Wilson et al., 2006). By contrast, SIRT3, SIRT4, and SIRT5 are primarily found in the mitochondria. SIRT3 has the capability to shuttle from the nucleus to the mitochondria via its mitochondrial localization sequence, which is also responsible for its mitochondrial deacetylation activity (Onyango et al., 2002; Schwer et al., 2002; Lombard et al., 2007; Bao et al., 2010). Of note, these mitochondrial regulators transfer to the nucleus in response to DNA damage induced by etoposide treatment or ultraviolet (UV) irradiation (Scher et al., 2007). SIRT6 is widely distributed, being found in the nuclear plasma, the heterochromatin, the nucleolus, as well as the cytoplasm (Michishita et al., 2005; Ardestani and Liang, 2012; Jedrusik-Bode et al., 2013). SIRT6 has a slower catalytic rate than other active SIRTs on substrates because SIRT6 lacks the conserved, highly flexible NAD^+^-binding loop, and instead contains a stable single helix (Pan et al., 2011). Finally, SIRT7, predominantly locates in the nucleolus but also exists in the cytoplasm (Nahalkova, 2015; Zhang et al., 2016) and is involved in mitochondrial function (Ryu et al., 2014; Mohrin et al., 2015). Two sequences in the N-terminal and C-terminal regions of SIRT7 permit nuclear and nucleolar localization, respectively (Kiran et al., 2013).

#### Class IV HDACs

HDAC11 is the exclusive member of the class IV HDACs. Recent studies have indicated that HDAC11 might predominantly be more involved in the fatty acylation of proteins compared to its weak deacetylation (Kutil et al., 2018; Cao et al., 2019).

Here, we recapitulate the detailed distributions of all 18 HDACs in Table 2.

**TABLE 2:** Some phenotypes observed after some manipulations of HDACs in different models.

HDACs	Subcellular location	Knockout and knockdown models	References
HDAC1	Cytoplasm; Nucleus (nucleoplasm, heterochromatin)	Accelerates tumor development in skin tumors Promotes p21-mediated cell cycle arrest in mouse embryonic fibroblasts (MEFs)	Winter et al., 2013 Zupkovitz et al., 2010
HDAC2	Cytoplasm; Nucleus (nucleoplasm, heterochromatin)	HDAC1/2 double KO (HD1/2DKO) disrupts mitotic progress, chromosome segregation and causes loss of cell viability in embryonic stem cell (ESC) HD1/2DKO represses Myc- and p53- associated tumorigenesis in lymphomas HD1/2DKO induces apoptosis in thyroid cancers HD1/2DKO causes nuclear fragmentation and mitotic catastrophe HD1/2DKO affects CD4^+^ T cell lineage differentiation Skeletal muscle-specific HD1/2DKO causes autophagy blockage-associated abnormal metabolism and perinatal lethality of mice HD1/2DKO downregulates T-cell receptor (TCR) signaling pathway and neoplastic transformation of immature T cells	Jamaladdin et al., 2014 Heideman et al., 2013 Lin et al., 2019 Haberland et al., 2009 Boucheron et al., 2014; Preglej et al., 2020 Montgomery et al., 2007; Moresi et al., 2012; Dovey et al., 2013
HDAC3	Plasma Membrane; Cytoskeleton (mitotic spindle); cytoplasm; Golgi apparatus; Nucleus (nucleoplasm)	Global deletion of HDAC3 causes embryoni lethality of mice; cardiac-specific deletion of HDAC3 shows only 3−4 months survival of mice accompanying with cardiac metabolic disorder and mitochondrial dysfunction Represses hepatocellular carcinoma (HCC), multiple myeloma (MM) proliferation and growth Induces genome instability, cell cycle arrest and apoptosis Disrupts DNA damage repair in HCC Affects T cell maturation Represses prostate tumorigenesis and progression Stimulates rhabdomyosarcoma differentiation and limits tumor growth in presence of tamoxifen Represses inflammatory response	Montgomery et al., 2008 Lu et al., 2018; Ho et al., 2020 Bhaskara et al., 2008; Bhaskara et al., 2010; Jiang and Hsieh, 2014 Ji et al., 2019 Hsu et al., 2015 Yan Y. et al., 2018 Phelps et al., 2016 Chen et al., 2012
HDAC8	Plasma Membrane; Cytoplasm; Nucleus (nucleoplasm, chromosome)	Induces p53-dependent hyperactivation of apoptosis	Hua et al., 2017
HDAC4	Cytoskeleton (actomyosin); Cytoplasm; Nucleus (nucleoplasm)	Causes mitotic arrest and chromosome segregation Causes partial proliferation deficit in leiomyosarcomas Impairs type I IFN signaling and causes spread of DNA virus Stimulates chondrocyte differentiation Promotes cell growth of myelodysplastic syndrome (MDS) or AML Causes reduced exercise capacity, cardiac dysfunction and heart failure	Cadot et al., 2009 Di Giorgio et al., 2020 Lu Y. et al., 2019 Nishimori et al., 2019 Huang et al., 2020 Lehmann et al., 2018; Kronlage et al., 2019
HDAC5	Cytoplasm; Golgi apparatus; Nucleus (nucleoplasm)	Impairs CD8^+^ T-cell IFN-γ production in lung adenocarcinoma Promotes HDAC2-dependent hypertrophic stresses Stimulates chondrocyte differentiation HDAC4/5DKO confers resistance to muscle proteolysis and atrophy	Xiao et al., 2016 Eom et al., 2014 Nishimori et al., 2019 Moresi et al., 2010
HDAC7	Cytoplasm; Nucleus (nucleoplasm)	Blocks early B-cell development	Azagra et al., 2016
		Affects thymocytes cell survival and thymic T cell development.	Kasler et al., 2011
		Causes loss of vascular integrity and embryonic lethality	Chang et al., 2006
		Stimulates β-catenin-dependent proliferation of chondrocytes	Bradley et al., 2015
		Abrogates growth of lung cancer	Lei et al., 2017
HDAC9	Cytoplasm; Nucleus (nucleoplasm)	Exhibits stress-dependent cardiac hypertrophy	Zhang et al., 2002a
		Accelerates adipogenic differentiation	Chatterjee et al., 2011
		Decreases CD8^+^ dendritic cell infiltration	Ning et al., 2020
		Decreases cell adhesion and migration, promotes apoptosis and dramatically impairs proliferation in leiomyosarcomas	Di Giorgio et al., 2020
HDAC6	Plasma membrane; Cytoskeleton (microtubule); Cytoplasm; Aggresome; Endosome; Nucleus (nucleoplasm)	Induces interleukin-10 associated inflammatory response	Wang B. et al., 2014
		Affects immune response moderately	Zhang et al., 2008
		Represses endothelial cell migration and angiogenesis	Kaluza et al., 2011
		Blocks autophagy flux and tumorigenesis of Myc-driven neuroblastoma or KRAS- driven colorectal cancer (CRC) and MM	Kaliszczak et al., 2018
		Confers susceptibility to RNA virus infections	Choi et al., 2016
		Impairs actin cytoskeleton-dependent cell migration	Gao et al., 2007
HDAC10	Cytoplasm; Nucleus (nucleoplasm)	Promotes G2-M transition arrest in non-small cell lung cancer (NSCLC)	Li et al., 2015
		Interrupts autophagic flux in neuroblastoma cells	Oehme et al., 2013
		Activates chaperone-mediated autophagy (CMA) in HeLa cells	Obayashi et al., 2020
		Activates the TGF-β pathway in lung adenocarcinoma cells	Li et al., 2020
SIRT1	Cytoplasm; Mitochondrion; Nucleus (nuclear membrane, nucleoplasm, euchromatin, heterochromatin, nucleolus)	Impairs genome stability; embryonic lethality	Vaquero et al., 2007; Wang et al., 2008
		Represses angiogenesis	Potente et al., 2007; Dioum et al., 2009; Lim et al., 2010
		Impairs nicotinamide mononucleotide (NMN)-induced amelioration of liver fibrosis and NMN-dependent telomere integrity in premature aging mice	Amano et al., 2019
		Causes methionine restriction-induced lethality in mouse ESC	Tang S. et al., 2017
		Impairs myeloid-derived suppressor cells (MDSC) differentiation by disturbing glycolytic pathway	Liu et al., 2014
		Impairs various DNA repair	Cohen et al., 2004; Yuan et al., 2007; Ming et al., 2010; Meng et al., 2020
		Inhibits autophagy in MEFs	Lee et al., 2008
		Causes differentiation defects of mice ESC	Tang et al., 2014
		Suppresses BCR-ABL transformation and chronic myelogenous leukemia (CML) proliferation	Yuan et al., 2012
		Reduces both B-cell and plasma cell differentiation and prevents graft-versus-host disease (GVHD)	Daenthanasanmak et al., 2019
SIRT2	Plasma membrane; Cytoskeleton (centriole, centrosome, microtubule, meiotic spindle, mitotic spindle); Cytoplasm; Mitochondrion; Nucleus (nucleoplasm, chromosome, telomeric region)	Decreases breast cancer cell viability	Jing et al., 2016
		Causes genomic instability and chromosomal aberration in skin squamous cell carcinoma	Serrano et al., 2013
		Promotes NHEJ and HR repair under irradiation	Nguyen et al., 2019
		Suppresses angiogenesis in CRC	Hu et al., 2018
		Inhibits glycolysis and tumor growth in breast cancer	Park et al., 2016
		Increases migration and invasion and decreases sensitivity of oxidative stress upon radiation	Nguyen et al., 2014
		Disturbs type I IFN signaling gene transcription and inhibits CDK9-associated proliferative signaling	Kosciuczuk et al., 2019
SIRT3	Cytoplasm; Mitochondrion and mitochondrial matrix; Nucleus (nucleoplasm)	Inhibits SHMT2-involved serine disorder in CRC proliferation	Wei et al., 2018
		Augments ROS generation and HIF-1α-involved glycolysis in breast cancer	Finley et al., 2011
		Induces abnormal mitochondrial physiology, oxidative stress and genomic instability	Kim et al., 2010
		Reduces ROS production in GVHD	Toubai et al., 2018
		Promotes colon sensitivity to inflammation and tumorigenesis of CRC	Zhang et al., 2018
		Inhibits Complex I and Complex II activity of the electron transport chain; reduces mitochondrial membrane potential and impairs mitochondrial homeostasis	Ahn et al., 2008; Cimen et al., 2010; Yang et al., 2016
		Enhances glycometabolism-associated proliferation of cholangiocarcinoma	Xu et al., 2019
		Promotes ROS production, glycolysis, cell transformation and tumorigenesis of breast cancer	Zou et al., 2017
		Induces metabolic disorder, autophagy and cell death in diffuse large B-cell lymphoma (DLBCL)	Li M. et al., 2019
SIRT4	Mitochondrion (mitochondrial inner membrane, mitochondrial matrix)	Upregulates amino acid-stimulated insulin secretion in insulinoma cells or other tissues	Haigis et al., 2006; Anderson et al., 2017
		Suppresses anabolic metabolism, autophagy and cell proliferation	Shaw et al., 2020
		Accelerates lymphomagenesis of Myc-induced Burkitt lymphoma and promotes glutamine metabolism	Jeong et al., 2014
		Attenuates hepatic steatosis	Guo et al., 2016
		Increases glutamine-dependent proliferation, stress-induced genomic instability in lung cancer	Jeong et al., 2013
SIRT5	Cytoplasm; Mitochondrion; Nucleus	Suppresses glutamine metabolism-associated tumor proliferation	Greene et al., 2019
		Downregulates SHMT2-involved serine metabolism and delays tumor cell growth	Yang et al., 2018
		Increases oxidative DNA damage	Chen et al., 2018
		Decreases NADPH production; Increases ROS production and susceptibility to oxidative stress	Zhou et al., 2016
		Disturbs Braf^*V600E*^-mediated cutaneous melanoma formation and growth	Moon et al., 2019
		Decreases ATP production and activates AMP-activated protein kinase (AMPK) to attenuate cardiac hypertrophy of mice; heart-specific SIRT5 KO induces oxidative stress and cardiac hypertrophy	Hershberger et al., 2018; Zhang et al., 2019
SIRT6	Cytoplasm; Nucleus (nucleoplasm, nuclear telomeric heterochromatin, nucleolus)	Enhances aerobic glycolysis and MYC-driven tumor growth in colorectal cancer and pancreatic cancer	Sebastian et al., 2012
		Induces KRAS- and Lin28b-driven tumorigenesis of pancreatic ductal adenocarcinoma (PDAC)	Kugel et al., 2016
		Promotes FoxO1-dependent gluconeogenesis in CRC	Zhang et al., 2014
		Upregulates HIF-1α-induced glycolysis	Zhong et al., 2010
		Inhibits PPARα signaling transcription	Naiman et al., 2019
		Induces metabolism- and oncogene-driven hepatocarcinogenesis	Marquardt et al., 2013
		Inhibits cell proliferation and survival of skin carcinoma	Ming et al., 2014
		Induces genome instability and sensitivity to genotoxic damage	Toiber et al., 2013
		Promotes HR and NHEJ repair	Hou et al., 2020; Rezazadeh et al., 2020
		Senses the DDR	Onn et al., 2020
		Upregulates the IFN pathway	Simon et al., 2019
		Impairs differentiation in mESC and human embryoid body (hEB)	Etchegaray et al., 2015
SIRT7	Cytoplasm; Nucleus (nucleoplasm, heterochromatin, nucleolus)	Upregulates HIF-1α and HIF-2α transcriptional activity	Hubbi et al., 2013
		Activates TGF-β	Tang X. et al., 2017
		Increases replication stress and impairs NHEJ repair	Vazquez et al., 2016
		Represses cell cycle arrest	Lu Y. F. et al., 2020
		Upregulates cGAS-STING pathway	Bi et al., 2020
		Causes LINE-1-associated genome instability and compromised viability	Vazquez et al., 2019
		Impairs Sirt1-PPARγ-dependent adipogenesis and adipocyte differentiation	Fang et al., 2017
		Inhibits the proliferation and invasion in thyroid cancer	Li H. et al., 2019
HDAC11	Plasma membrane; Cytoplasm; Nucleus (nucleoplasm)	Enhances type I IFN signaling	Cao et al., 2019
		Enhances proinflammatory cytokine production, proliferation of T cells and GVHD	Woods et al., 2017
		Confers a metabolic homeostasis disorder	Bagchi et al., 2018; Sun et al., 2018
		Suppresses JAK2-driven proliferation and survival of myeloproliferative neoplasm (MPN)	Yue et al., 2020
		Suppresses lymph node metastases in breast cancer	Leslie et al., 2019

## Biological Functions of HDACs

Histone deacetylases are expressed in different tumors: class I and II HDACs are considered to be general oncoproteins that interact with substrates and regulate gene expression to promote tumorigenesis and cancer development either individually or alongside with co-repressors (Falkenberg and Johnstone, 2014; West and Johnstone, 2014). Paradoxically, SIRTs can serve as both oncoproteins and tumor suppressors (Kugel et al., 2016; Costa-Machado et al., 2018; Funato et al., 2018). We list the demonstrated knockout (KO) or knockdown models of 18 HDACs (Table 2). Because of the diverse biological function of HDACs, it is not surprising that HDACi regimens influence many cellular processes, including those that contribute to cancer progression.

### Transcriptional Regulation

#### Transcription Modulators

Transcription factors (TFs) can either directly target DNA or undergo various PTMs to alter gene expression. In this manner, HDACs negatively modulate transcription through forming a complex with TFs or by directly regulating TF transcription (Grunstein, 1997). For instance, the v-myc avian myelocytomatosis viral oncogene homolog (Myc) is a well-characterized proto-oncogene that promotes tumorigenesis by directly recruiting and interacting with HDACs to regulate gene expression (Liu et al., 2007; Zhang et al., 2012a). Meanwhile, Myc acetylation is also modulated by HDACs either directly or indirectly. For example, SIRT2 stabilizes N-Myc and c-Myc proteins by deacetylating and repressing neuronal precursor cell-expressed developmentally downregulated 4 (NEDD4), which mediates Myc ubiquitination and degradation (Liu et al., 2013). Consequently, the SIRT2-specific inhibitor thiomyristoyl (TM) promotes Myc ubiquitination and degradation (Jing et al., 2016). HDACi suberoylanilide hydroxamic acid (SAHA) and entinostat (also called MS-275) induce Myc acetylation at K323, downregulating Myc and accompanying with tumor necrosis factor (TNF)-related apoptosis-inducing ligand (TRAIL) activation (Nebbioso et al., 2017). Therapeutic regimens that target Myc suppression using HDACi combined with DNA demethylation reagents seems to have a notable effect on non-small cell lung cancer (NSCLC) through activating immune system (Topper et al., 2017).

p53 is a well-known TSG that is crucial for mediating gene expression (Gu and Zhu, 2012; Zhu, 2017): its activity is modulated by various PTMs. HDACs and SIRTs downregulate p53 activity to promote cancer cell survival in response to oxidative stress (Juan et al., 2000; Luo et al., 2000, 2001; Vaziri et al., 2001). Specifically, HDAC1, -2, and -3 all can induce p53 deacetylation that represses p53-mediated apoptosis (Juan et al., 2000). In addition, HDAC2 modulates p53 transcriptional activity through direct p53-DNA binding (Harms and Chen, 2007). p53 binds to DNA depending on its acetylation state at K373/K382 by p300 (Gu and Roeder, 1997). HDACi depsipeptide induces acetylation at K373/K382 by recruiting p300. This in turn promotes the expression of p21^*Cip1*/*Waf1*^ (encoded by *cyclin dependent kinase inhibitor 1A, CDKN1A*) (Zhao et al., 2006). Compared to wild type p53, HDAC deficiency reduces mutant p53 (mtp53) expression both at the mRNA and protein level (Yan et al., 2013; Stojanovic et al., 2017). Besides the transcriptional regulation of mtp53, HDACs also modulate mtp53 protein stability. By inhibiting HDAC6, SAHA promotes the preferential degradation of mtp53 by downregulating heat shock protein 90 (HSP90) that suppresses p53 degradation via E3 murine double minute (MDM2) or carboxy terminus of HSP70-interacting protein (CHIP) (Li et al., 2011). Therefore, inducing mtp53 degradation by blocking HDAC6-HSP90 might represent a novel strategy to suppress oncogenesis in the future (Alexandrova et al., 2015).

In addition to TFs, HDACs also modulate the activity of super enhancers (SEs) (Gryder et al., 2019). Enhancer RNAs (eRNAs) are short, non-coding RNA molecules that alter the transcription of target genes in cooperation with promoters (Melo et al., 2013; Hsieh et al., 2014; Danko et al., 2015; Mao et al., 2019). Trichostatin A (TSA) and SAHA reduce eRNA synthesis by inhibiting HSP90 (Greer et al., 2015). MEF2D and HDAC4/9 form a corepressor to recognize intergenic regions. HDAC4/9 depleted cells show increased H3K27ac level around the gene transcriptional start sites where show the features of active enhancers within corresponded topologically associated domains (TAD) (Di Giorgio et al., 2020). Class I-specific HDACi 4SC-202 globally increases both of H3K27ac and H3K4me3 levels around the TSS of genes, but notably decreases occupancy at proximal regions of TSS of genes such as *SMAD family member 6 (SMAD6)* and *E2F transcription factor 8 (E2F8)*, which are associated with enhancer deactivation (Mishra et al., 2017). Panobinostat and romidepsin alter the acetylation status of H3K27 by disrupting the SE topology in *paired box 8* (*PAX8*) (Shi et al., 2019). Largazole (a cyclic peptides similar to depsipeptide) preferentially disturbs SE-driven transcripts that are frequently associated with oncogenic activities (Sanchez et al., 2018).

#### Transcriptional Activation

Although HDACs generally function as gene silencers, they can also activate transcription (Kurdistani et al., 2002; Wang et al., 2002). Besides the regulation of enhancers, a potential mechanism underlying this role includes the modulation of RNA polymerase II (RNAP2) by HDACs. HDACs participate in the crosstalk between RNAP2 C-terminal domain acetylation and phosphorylation (Wang et al., 2009; Blank et al., 2017; Ali et al., 2019). SIRT6 recruits and mono-ADP-ribosylates switch/sucrose non-fermenting (SWI/SNF) related, matrix associated, actin dependent regulator of chromatin subfamily c member 2 (SMARCC2/BAF170) to form active chromatin at the enhancer of heme oxygenase-1, which subsequently recruits RNAP2 (Rezazadeh et al., 2019). SIRT6 can also bind p53 to effectively recruit RNAP2 to local promoters (Li et al., 2018). SIRT6 deficiency mediates the activation of cyclin-dependent kinase 9 (CDK9) that can phosphorylate negative elongation factor (NELF) and mediate NELF release from RNAP2, facilitating the enrichment of TFs and RNAP2-related elongation factors to promote elongation of specific gene sets (Etchegaray et al., 2019). Consistently, TSA and SAHA disturb RNAP2-mediated transcriptional elongation by promoting the association between RNAP2 and NELF (Greer et al., 2015). Moreover, high doses of largazole can cause RNAP2-mediated transcriptional pausing and cell death (Sanchez et al., 2018).

#### DNA Methylation and Deacetylation

DNA methylation and histone modification modulate transcription, either alone or cooperatively, by altering chromatin status. HDACs and their complexes are recruited to hyper-methylated DNA through methyl-CpG binding domain containing (MBD) protein (MeCP), which has transcriptional repression roles (Jones et al., 1998; Nan et al., 1998; Ng et al., 1999; Zhang et al., 1999; Zhu et al., 2003). To maintain DNA methylation, DNA (cytosine-5-)-methyltransferase 1 (DNMT1) binds to HDAC1 and HDAC2 to establish heritable transcriptional silencing (Robertson et al., 2000; Rountree et al., 2000). A number of major breakthroughs involving combinations of HDACi and DNA demethylation reagents [DNA methyltransferase inhibitors (DNMTi)] have occurred in the past two decades (Cameron et al., 1999; Zhu et al., 2001b; Topper et al., 2017). The rationale underlying combined DNMTi and HDACi therapy lies in their synergistic effects on compacted chromatin. Dense methylation of CpG islands (CGI) is responsible for silencing genes, which can be reactivated by HDACi. In this scenario, TSA loosens the structure of chromatin and induces the expression of previously silenced genes in the presence of DNMTi (Jones et al., 1998, 2016; Cameron et al., 1999). The combination of 5-aza-2’-deoxycytidine (5-Aza-CdR, decitabine, Dacogen, Otsuka) and either depsipeptide or TSA induces the expression of p21^Cip/Waf1^, p15 (*CDKN2B/INK4B*), p16 (*CDKN2A/INK4B*), and p19 (*CDKN2D/INK4D*) (Cameron et al., 1999; Zhu et al., 2001a). Depsipeptide and apicidin induce demethylation and re-activate silenced genes such as p16, GATA binding protein 4 (GATA4) and sal-like protein 3 (SALL3) by inhibiting DNMT1 binding to these gene promoters (Wu et al., 2008). In terms of the direct anti-tumor effects, DNMTi in combination with HDACi can provoke a durable, powerful clinical response in patients (Jones et al., 2016). Several combinational therapies present in regimen of reversing tumor immune evasion in NSCLC. Azacytidine plus ITF-2357 (givinostat) seems to be the most efficient strategy that augments antigen presentation machinery and interferon α/β (IFNα/β)-related immune gene activation, and mainly focuses on suppression of Myc-driven tumorigenesis (Topper et al., 2017). With their broad range of physiological functions in various tissues, the combined effects of HDACi and DMNTi hold substantial therapeutic promise going forward.

#### Synergetic Regulation of HDACs and Other Histone Modifiers

In addition to DNA methylation, HDACs also cooperate with other epigenetic modifiers. For example, lysine-specific demethylase 1 [LSD1, lysine demethylase 1A (KDM1A)] is responsible for removing mono- or di-methylation of H3K4, and represses transcription via the CoREST-HDACs complex (Humphrey et al., 2001; Lee et al., 2005; Shi et al., 2005). A dual inhibitor of HDAC and LSD1, corin, has been developed to suppress CoREST-HDACs and to coordinately increase H3K4me1, H3K27ac and H3K27me3 (Kalin et al., 2018; Anastas et al., 2019). KDM2B induces H3K79 demethylation and transcriptional repression in a SIRT1-dependent manner (Kang et al., 2018). KDM4A modulates gene repression though a physiological interaction with the NCoR-HDAC3 complex (Zhang et al., 2005). KDM5A directly associates with HDAC complexes to regulate H3K4me2/3 (Nishibuchi et al., 2014). The H3K36me2 demethylase KDM8 increases H3/H4 acetylation and Cyclin A1 transcriptional activation by impeding HDAC1 recruitment (Hsia et al., 2010).

Regarding histone lysine methyltransferases, HDAC3 modulates the H3K9ac/H3K9me3 transition in a suppressor of variegation 3-9 homolog 1 (SUV39H1, also called KMT1A)-dependent manner during the DNA damage response (DDR) (Ji et al., 2019). SIRT1 regulates H3K9 methylation by deacetylating K266 in the Su(var)3-9, enhancer-of-zeste and trithorax (SET) domain of SUV39H1, thus increasing its activity during heterochromatin formation (Shankaranarayana et al., 2003; Vaquero et al., 2007). While SIRT6 induces the monoubiquitination of cysteines (Cys) in the pre-SET domain of SUV39H1, removing SUV39H1 from IκBα negatively regulates the nuclear factor-kappaB (NF-κB) pathway (Santos-Barriopedro et al., 2018). Depsipeptide decreases H3K9me2/3 expression by reducing the expression of SUV39H1 and G9A (also called KMT1C) (Wu et al., 2008). SIRT2 binds and deacetylates PR-Set7/SET8/KMT5A at K90, and increases the H4K20me1 level (Serrano et al., 2013). HDACs also interact with polycomb-group (PcG) proteins to reset chromatin remodeling and transcriptional repression (Van Der Vlag and Otte, 1999; Kuzmichev et al., 2005; Zhang et al., 2012b; Fukumoto et al., 2018). SIRT1 interacts with Set7/9 (also called KMT7), with several sites being methylated by Set7/9. In response to DNA damage, SIRT1-p53 binding is significantly enhanced in the presence of Set7/9 and this binding coincide with increased p53 acetylation at K382 (Liu et al., 2011).

**FIGURE 2:**
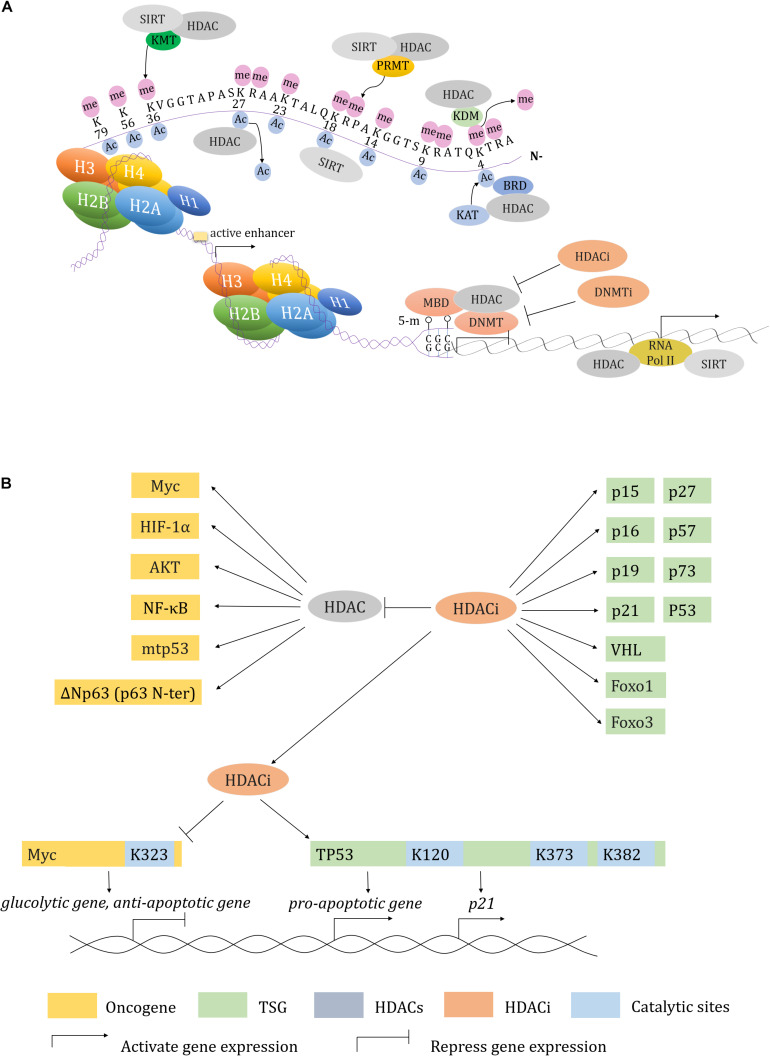
Transcription regulation in HDACs and HDACi. **(A)** HDAC and HDACi involved transcription regulation in concert with other epigenetic modifiers. **(B)** A working model described how HDAC and HDACi regulate both of oncogenes and tumor suppressor genes expression.

The BRD family proteins are readers of Kac (Dhalluin et al., 1999; Fujisawa and Filippakopoulos, 2017). Class I HDACi 4SC-202, mocetinostat and entinostat induce increase of hundreds of gene expression, which are mostly enriched upon BRD4- and MYC-targeted TSS-proximal regions. p21 is activated by 4SC-202 to inhibit cell proliferation (Mishra et al., 2017). Similarly, JQ1 cooperates with SAHA to inhibit the growth of pancreatic ductal adenocarcinoma (PDAC) by upregulating p57 (*CDKN1C*) that usually blocked by Myc (Mazur et al., 2015). Besides, HDACs also interact with protein arginine methyltransferases (PRMTs) to regulate gene transcription (Qi et al., 2018; Yan W. W. et al., 2018). These findings all highlight the competition among the “readers”, “writers” and “erasers” at acetylated histones and non-histones, and may provide additional, novel and combination epigenetic approaches for cancer therapy in the future (Figure 2).

### Metabolism

Various kinases and metabolic pathways form a complex network with epigenetic co-repressors to dynamically regulate metabolic flux and enzyme activity; aberrations in these processes can result in tumorigenesis and cancer progression. Metabolism can affect protein acetylation by altering the concentration of NAD^+^ and acetyl-CoA. In turn, HDACs also mediate metabolic reprogramming in cancer cells (Verdin and Ott, 2015).

Cancer cells are often characterized by their strong glycolytic activity, with aerobic glycolytic activity being preferred for tumor energy metabolism (Weinhouse, 1956). Increased glycolysis is associated with the abnormal regulation of glycolytic enzymes and other glucose metabolism pathways. Class II HDACs induce trans-repression of gluconeogenic enzymes from the cytoplasm to the nucleus in an HDAC3-dependent manner, and mediate the deacetylation and activation of the forkhead box class O (FoxO) family in the nucleus (Mihaylova et al., 2011). SIRT2 deacetylated isocitrate dehydrogenase 1 (IDH1) at K224 and promotes IDH1 enzymatic activity. The hypoacetylated IDH1 converts isocitrate into α-ketoglutarate in the tricarboxylic acid (TCA) cycle to inhibit liver metastases of colorectal cancer (CRC) (Wang B. et al., 2020). Pyruvate kinase (PKM2) promotes tumorigenesis by regulating oncogene expression and proliferation pathway activation in HDAC3-dependent way (Yang W. et al., 2011; Yang et al., 2012). SIRT6 can directly interact with and deacetylate PKM2, resulting in its nuclear export via exportin 4 and suppression of PKM2-related oncogenic functions (Bhardwaj and Das, 2016). Conversely, SIRT3 and SIRT6 also act as tumor suppressors, restricting aerobic glycolysis in cancer cells through destabilization of hypoxia inducible factor 1 subunit alpha (HIF-1α) and inhibition of glycolytic kinases, respectively (Finley et al., 2011; Sebastian et al., 2012). p53 directly binds and activates SIRT6 to regulate gluconeogenesis by mediating the nuclear exclusion and deacetylation of FoxO1 (Zhang et al., 2014).

The fatty acylation of proteins has a vital role in membrane synthesis, vesicle transport, protein-membrane interaction, cell signaling and localization (Resh, 2006). HDACs regulate fatty acylation during cancer progression. For example, HDAC8 performs lysine de-fatty-acylation functions. The HDAC8-selective inhibitor PCI-34051 also increases overall fatty acylation levels in Jurkat cells (Aramsangtienchai et al., 2016). HDAC11 has a relatively low effect on acetyl groups, but efficiently catalyzes dodecanoylated and myristoylated peptides (Kutil et al., 2018). Compared with acetyl peptides, some HDACs have higher catalytic efficiency on acyl groups (Houtkooper et al., 2012; Feldman et al., 2013; Jiang et al., 2013; Aramsangtienchai et al., 2016; Wang et al., 2016; Kutil et al., 2018) (see Table 1). HDAC11 efficiently removes acyl groups on the surface of serine hydroxymethyltransferase 2α (SHMT2α), causing SHMT2α dissociation from the late endosome/lysosome. This effect leads to type I interferon receptor chain 1 (IFNαR1) polyubiquitylation and degradation, as well as downregulation of IFN signaling (Cao et al., 2019). HDAC11-specific inhibitors, such as elevenostat, FT895, and SIS17, might represent promising future treatments that target lipid metabolic dysregulation in cancers (Martin et al., 2018; Kutil et al., 2019; Son et al., 2019). SIRT3 has a role in mitochondrial fatty-acid β-oxidation by regulating long-chain acyl-CoA dehydrogenase (LCAD) (Hirschey et al., 2010). SIRT6 is indispensable for hepatic β-oxidation by deacetylating the peroxisome proliferator-activated receptor α (PPARα) coactivator nuclear receptor coactivator 2 (NCOA2) at K780 (Naiman et al., 2019). Following palmitic acid treatment, SIRT6 interacts with p53 to regulate *de novo* cardiolipin biosynthesis and maintain lipid homeostasis (Li et al., 2018).

Amino acids are also involved in tumorigenesis. SIRT3 depletion suppresses glutamate dehydrogenase (GDH/GLUD), which impairs glutamine flux to the TCA cycle and causes reduction of acetyl-CoA pools (Li M. et al., 2019). SIRT4 is a lipoamidase that diminishes the activity of the pyruvate dehydrogenase complex (PDH) by hydrolyzing the lipoamide cofactor dihydrolipoyllysine acetyltransferase (DLAT) (Mathias et al., 2014). Furthermore, SIRT4 represses GDH activity through its ADP-ribosyltransferase function. SIRT4 deficiency activates GDH, stimulating amino acid-mediated insulin secretion in insulinoma cells (Haigis et al., 2006). SIRT4 also mediates other PTMs, including methylglutarylation, hydroxymethylglutarylation and 3-methylglutaconylation, and intermediates of these PTMs contribute to leucine oxidation. Indeed, SIRT4-KO induces leucine disordered metabolism and leads to glucose intolerance and insulin resistance (Anderson et al., 2017). Meanwhile, elevated SIRT5 expression in breast cancer mediates glutaminase desuccinylation and protects glutaminase from ubiquitin-mediated degradation; this effect has been associated with a poor prognosis in breast cancers (Greene et al., 2019). SIRT3 and SIRT5 also mediate desuccinylation and deacetylation of SHMT2, respectively, suggesting that suppression of serine catabolism might represent a novel strategy to restrain tumor growth (Wei et al., 2018; Yang et al., 2018).

### Hypoxia and Angiogenesis

Activated HIFs (HIF-1α, HIF-2α, HIF-3α, and HIF-1β) have vital roles in adaptive responses, with HIF-1α and HIF-2α in particular being associated with tumorigenesis and angiogenesis in response to hypoxia (Gonzalez et al., 2018). Notably, SAHA specifically induces the accumulation of HIF-2α rather than HIF-1α in soft tissue sarcomas (Nakazawa et al., 2016). HIF-1α is ubiquitinated by von Hippel-Lindau (VHL) or by binding to p53-MDM2, inducing proteasomal dependent degradation (Vriend and Reiter, 2016; Gonzalez et al., 2018). HDAC1 downregulates p53 and VHL expression, and stimulates HIF-1α-dependent angiogenesis. TSA inhibits this process by blocking HIF-1α and the vascular endothelial growth factor (VEGF) receptor (Kim et al., 2001). Besides, HDAC4 and HDAC6 directly bind to HIF-1α. HDACi LAQ824, valproic acid (VPA) and trapoxin induce dose-dependent HIF-1α depletion in an VHL-independent manner (Qian et al., 2006). The class IIa-selective HDACi TMP195 effectively establishes an anti-tumor microenvironment and induces normalization of tumor vasculature in breast cancers by eliciting recruitment and differentiation of macrophages. TMP195 in combination with chemotherapeutic regimens such as carboplatin or paclitaxel can significantly reduce breast cancer burden (Guerriero et al., 2017).

As for SIRTs, they continuously perform an inhibitory role to HIF-1α-relevant transcriptional and metabolic regulation. During hypoxia, SIRT1 activity is inhibited due to reduced NAD^+^ levels, which leads to the acetylation and activation of HIF-1α and HIF-2α. SIRT1 negatively regulates angiogenesis by deacetylating FoxO1 (Potente et al., 2007; Dioum et al., 2009; Lim et al., 2010). In human breast cancers, a SIRT3 deficiency can stabilize HIF-1α (Finley et al., 2011). Both SIRT6 and SIRT7 can negatively modulate the expression and activity of HIF-1α and HIF-2α (Zhong et al., 2010; Hubbi et al., 2013).

### Redox and Oxidative Stress

Histone deacetylase inhibitors treatment is often accompanied by oxidative stress related DNA damage that is primarily caused by the generation of reactive oxygen species (ROS) (Xu et al., 2006). In mammalian cells, two redox systems respond to oxidative stress: the thioredoxin (Trx) system and the glutathione-glutaredoxin (Grx) system. In response to nitric oxide (NO), HDAC2 is S-nitrosylated at Cys 262 and Cys 274, which induces chromatin remodeling to promote gene expression (Nott et al., 2008). A pair of redox-sensitive cysteine residues (Cys-667/Cys-669) in HDAC4 are involved in oxidative stress via the formation of intramolecular disulfide bonds (Ago et al., 2008). Compared with normal cells, tumor cells are enriched with the antioxidant Trx reductase (TrxR), which might represent a novel therapeutic target (Lu and Holmgren, 2014; West and Johnstone, 2014). Depsipeptide causes robust DNA damage and apoptosis by inducing ROS generation, primarily through the suppression of TrxR (Wang et al., 2012). HDAC5 represses mitochondrial ROS generation, and depletion of HDAC5 provokes nuclear factor, erythroid 2 like 2 (NRF2)-associated transcription (Hu et al., 2019). The DNA and RNA binding protein Y-box binding protein 1 (YB-1) binds to NRF2 in response to oxidative stress. Entinostat induces YB-1 acetylation and blocks its binding to NRF2, reducing NRF2 synthesis and increasing ROS levels in sarcoma cells (El-Naggar et al., 2019).

**FIGURE 3:**
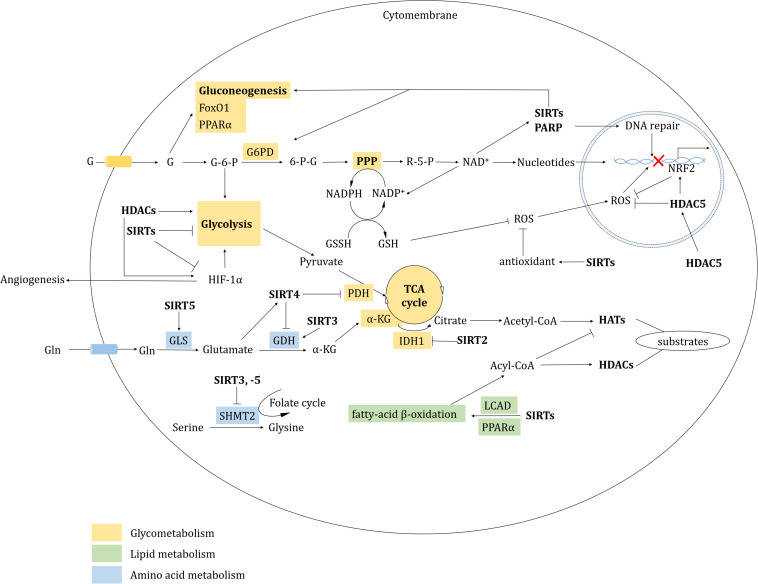
HDAC-involved metabolic regulation. HDACs regulate metabolism mainly including glycometabolism, lipid metabolism, amino acid metabolism and redox.

Sirtuins primarily serve as antioxidants in redox signaling. SIRT1, -2, and -3 all prevent oxidative stress by inducing or modulating manganese superoxide dismutase (MnSOD) (Brunet et al., 2004; Wang et al., 2007). Glucose-6-phosphate dehydrogenase (G6PD) is key enzyme of the pentose phosphate pathway (PPP) that regulates nicotinamide adenine dinucleotide phosphate (NADP)/NADPH levels. NADPH maintains glutathione (GSH) at a reduced state, which serves as an antagonist to prevent ROS generation (Chen et al., 2019). In response to oxidative stress, SIRT2 and SIRT3 promote NADPH generation by deacetylating and activating G6PD and IDH2 in the PPP or in the TCA cycle, respectively. The PPP also produces ribose-5-P, which synthesizes nucleotides and generates NAD^+^, which in turn supports SIRTs activity (Schlicker et al., 2008; Wang Y. P. et al., 2014). SIRT3 also activates NADH quinone oxidoreductase (Complex I) and succinate dehydrogenas (Complex II) in the electron transport chain (Ahn et al., 2008; Cimen et al., 2010). Furthermore, in the mitochondrial inter-membrane space, SIRT5 deacetylates cytochrome c (Schlicker et al., 2008). SIRT5 is also present in peroxisomes, where it desuccinylates and inhibits peroxisomal acyl-CoA oxidase 1 (ACOX1). A SIRT5 deficiency in hepatocellular carcinoma (HCC) increases oxidative DNA damage by elevating ACOX1-mediated H_2_O_2_ production (Chen et al., 2018). By contrast, SIRTs also inhibit antioxidation; for example, SIRT2 deacetylates and suppresses peroxiredoxin (an antioxidant) in breast cancer cells (Fiskus et al., 2016; Figure 3).

### DNA Damage Response

The DDR is a vitally important regulatory mechanism that protects genomic DNA from damage induced by various stimuli (Jackson and Bartek, 2009). Different levels of DNA damage are inevitably caused by UV radiation and DNA adducts, which are produced by ROS and reactive nitrogen species (RNS), as well as exposure to chemical agents (Barker et al., 2015; Roos et al., 2016; Pouget et al., 2018; Guo et al., 2020). DNA double-strand breaks (DSBs) are the most severe form of DNA damage and are repaired via one of two pathways: homologous recombination (HR) or non-homologous end-joining (NHEJ) (Scully et al., 2019). Deacetylation of H3K56 and H4K16 by HDAC1/2 are involved in mediating dynamic chromatin regulation in response to NHEJ (Miller et al., 2010). Although H4K16 acetylation attenuates binding of p53 binding protein 1 (53BP1) to H4K20me2, euchromatic histone lysine methyltransferase 1 (EHMT1, also called GLP or KMT1D)-catalyzed H4K16 monomethylation could significantly enhance this binding (Lu X. et al., 2019). Meanwhile, SIRT1 redistributes to DSB foci to promote HR during oxidative stress (Oberdoerffer et al., 2008). NuRD complex subunit, chromodomain-helicase-DNA-binding protein (CHD4), was recently discovered to be recruited by SIRT6 to replace heterochromatin 1 (HP1) at H3K9me3 to ultimately promote chromatin relaxation through HR (Hou et al., 2020). SIRT6 also mono-ADP-ribosylates and displaces KDM2A (also called JmjC domain-containing histone demethylase 1A, JHDM1A) from chromatin, which leads to HP1α-dependent H3K9me3 deposition at DSBs and transient transcriptional repression, accompanying with the recruitment of NHEJ factors (Rezazadeh et al., 2020).

The DDR is controlled by three related kinases: ataxia telangiectasia mutated (ATM), ataxia telangiectasia mutated and Rad3 related (ATR), and DNA-dependent protein kinase catalytic subunits (DNA-PKcs) (Blackford and Jackson, 2017). Once a DSB occurs, the MRE11-RAD50-NBS1 (MRN) complex and the Ku family are rapidly recruited to DSB sites. As the sensor, ATM is recruited to DSB sites by the MRN complex (Blackford and Jackson, 2017). The interplay between HDAC1 and ATM increases chromatin condensation to prevent radio-sensitivity in response to ionizing radiation (Kim et al., 1999). SIRT1 binds to deleted in breast cancer 1 (DBC1), which induces p53 activation (Kim et al., 2008; Zhao et al., 2008). ATM also mediates DBC1 phosphorylation at threonine (Thr) 454 which contributes to DBC1-SIRT1 interactions during DNA damage (Yuan J. et al., 2012). Moreover, SIRT1 deacetylates and maintains the hypoacetylation of Nijmegen breakage syndrome protein 1 (NBS1), which is necessary for the ionizing radiation-induced phosphorylation of NBS1 and subsequent MRN complex recruitment to DSB sites (Yuan et al., 2007). SIRT7 directly binds and deacetylates ATM, which is prerequisite for ATM dephosphorylation and inactivation in the final stage of DNA repair (Tang et al., 2019). Panobinostat-induced downregulation of meiotic recombination 11 homolog (MRE11) enhances radio-sensitization of bladder cancer cells by promoting MRE11 ubiquitination that relies on the upregulated E3 inhibitor of apoptosis protein 2 (cIAP2) (Nicholson et al., 2017).

ATR and its downstream kinase CHK1 are also involved in the response to DNA replication stress. SAHA slows down replication forks by restricting the ATR pathway (Conti et al., 2010). Following the conserved mechanism in yeast, VPA disrupts the formation of single-strand-DNA-RFA nucleofilaments and the activation of the Mec1 (ATR in human) and Rad53 (CHK2 in human) by suppressing the recruitment of replication factor A protein 1 [RFA1, replication protein A (RPA) in human] and DNA damage checkpoint protein Ddc2/LCD1 [ATR interacting protein (ATRIP) in human] to DNA damage sites (Robert et al., 2011). Entinostat represses checkpoint signaling during replication stress. Mechanically, HDAC1/2 suppress the cell cycle kinases WEE1 and CDK1 and induce the dephosphorylation of ATM and CHK2 by suppressing the expression of PP2A subunit. Entinostat also induces the incorrect incorporation of NTPs and metabolites during the induction of checkpoint kinase inactivation, which can result in mitosis catastrophe (Goder et al., 2018). Therefore, CHK inhibitors might be designed to prevent this event from occurring. Indeed, CHK1 inhibitor treatment combined with HDACi induces cell death via extensive mitotic disruption in a range of solid tumors (Lee et al., 2011).

DNA-PKcs is another sensor that is recruited to DSBs by Ku-bound DSB ends (Blackford and Jackson, 2017). Under conditions of fasting-induced oxidative stress, SIRTs act as protective factors in the DDR. Specifically, SIRT1 deacetylates Ku70, resulting in Ku70-Bcl-2 associated protein X (BAX) disassociation and the transport of BAX away from the mitochondria, leading to stress-induced resistance to apoptosis (Cohen et al., 2004). SIRT3 also physically interacts with and deacetylates Ku70 to impede BAX translocation to the mitochondria (Sundaresan et al., 2008).

SIRTs are highly important for DNA damage repair and genome stability (Tian et al., 2019; Ng and Huen, 2020). Recent data have shown that SIRT6 is a novel sensor for initiating the DDR (Onn et al., 2020). Deacetylated SIRT6 at K33 by SIRT1 results in SIRT6 polymerization and deposition at γH2AX foci. Moreover, a SIRT6 K33R hypoacetylation mimic can rescue DNA repair defects in SIRT1-deficient cancer cells (Meng et al., 2020). SIRT7 is also associated with NHEJ, as a Sirt7 deficiency impairs the recruitment of 53BP1 to DSB sites and inhibits NHEJ efficiency (Vazquez et al., 2016). SIRT7 also deacetylates ATM to mediate ATM inactivation in the final stage of DNA damage repair (Tang et al., 2019). SIRT7 acts as a deglutarylase to regulate H4K91 glutarylation (H4K91glu). This process is closely associated with chromatin remodeling in response to DNA damage (Bao et al., 2019). Treatment with 5-Fluorouracil (5-FU) induces SIRT7 degradation in the Tat-binding protein 1 (TBP1)-mediated proteasome-dependent pathway, increasing cell radiosensitivity in combination therapy (Tang M. et al., 2017).

Poly ADP-ribose polymerases (PARPs) are central to the activation of several downstream repair mechanisms, including single-strand DNA breaks (SSBs), base-excision repair (BER), HR and NHEJ (Pilie et al., 2019). SIRTs and PARPs all require NAD^+^ to elicit function. However, PARP1 consumes NAD^+^, and this affects NAD^+^-dependent SIRT activity. Thus, depleting PARP1 increases the catalytic function of SIRTs (Schreiber et al., 2006; Houtkooper et al., 2012; Imai and Guarente, 2014). Under conditions of oxidative stress, SIRT6 physically binds to and mono-ADP-ribosylates PARP1 at K521 to facilitate DNA repair (Mao et al., 2011). SIRT7 is recruited to DSB sites in a PARP1-dependent manner, and catalyzes H3K122 desuccinylation, which facilitates chromatin compaction and DNA repair (Li et al., 2016). Based on synthetic lethality, PARP inhibitors induce genomic instability in breast cancer susceptibility protein (BRCA1/2)-deficient cancer cells (Pilie et al., 2019). The combined use of HDAC and PARP inhibitors will likely be of great benefit for patients with BRCA1/2-deficient malignancies (Liszczak et al., 2018).

With the exception of DSBs, cancer cells can overcome DNA damage-induced cytotoxicity through BER, nucleotide excision repair (NER) and mismatch repair. Uracil-DNA N-glycosylase isoform 2 (UNG2) has a role in BER and can be deacetylated at K78 by HDAC, boosting disassociation from its E3 ubiquitin-like containing PHD and ring finger domain 1 (UHRF1) when stimulated by ROS. HDACi combined with genotoxic agents results in UNG2 degradation, resulting in a robust cell death effect (Bao et al., 2020). SIRT1 interacts with xeroderma pigmentosum group A (XPA) in NER by directly deacetylating XPA or mediating XPA binding to ATR to prevent UV irradiation (Fan and Luo, 2010; Jarrett et al., 2018). HDAC10 is mainly involved in DNA mismatch repair by deacetylating mutS homolog 2 (MSH2) at K73 (Radhakrishnan et al., 2015).

Besides, a number of other histone modifications are also actively involved in these DNA repair pathways, but are beyond the scope of this review (Cao et al., 2016; Kim J. J. et al., 2019; Li Z. et al., 2019). Suffice to say that the multiple sites of H3 and H4 acetylation are not absolutely related to checkpoint activation because the conversion of lysine to other amino acids can still activate checkpoints (Robert et al., 2011).

### Cell Cycle

Cell cycle dysregulation is a central hallmark of oncogenesis; as such, cell cycle regulators are considered promising targets for cancer treatment. HDACs are often involved in cell cycle checkpoints. HDAC3-mediated deacetylation of cyclin A affects the progression of the S phase and G2/M transitions (Bhaskara et al., 2008, 2010). HDAC10 depletion induces G2-M transition arrest through the regulation of cyclin A2. Mechanically, HDAC10 depletion induces the downregulation of high mobility group AT hook 2 (HMGA2), which leads to enrichment of E4F transcription factor 1 (a cyclin A2 repressor) at the cyclin A2 promoter and G2-M arrest (Li et al., 2015). Regarding combination therapies, the CDK9 inhibitor dinaciclib and panobinostat together induce apoptosis over the short-term in MLL-AF9-driven acute myeloid leukemia (AML) (Baker et al., 2016). The emerging hybrid inhibitor Roxyl-zhc-84, which concordantly inhibits HDACs and CDKs, induces G1-phase arrest and apoptosis in ovarian and breast cancer cells (Huang et al., 2018c).

Spindle assembly checkpoint (SAC) is involved in regulating mitosis. Budding uninhibited by benzymidazol related-1 (BubR1), a component of the SAC, must be deacetylated by HDAC2/3 to initiate mitotic exit (Park et al., 2017). HDAC3 induces SAC activation and the dissociation of sister chromatids (Eot-Houllier et al., 2008). SIRT2 is strongly associated with mitosis exit (Dryden et al., 2003). SIRT2 regulates the anaphase-promoting complex/cyclosome (APC/C) by deacetylating its cofactors, cell-division cycle protein 20 (CDC20) and CDC20 homolog 1 (CDH1), which are both required for mitosis exit and chromosome segregation (Kim et al., 2011). SIRT2 also deacetylates α-tubulin at K40 to promote cell mobility (North et al., 2003). The SIRT2 inhibitor SirReal2 induces tubulin hyperacetylation and BubR1 destabilization (Rumpf et al., 2015). HDAC5 induces the transcription of the mitosis kinase Aurora A, by repressing the expression of the E3 ligase NEDD4 (Sun et al., 2014). Combination of the Aurora A kinase inhibitor alisertib with romidepsin causes dose-dependent cytotoxicity of lymphoma cells (Zullo et al., 2015).

p21^Cip/Waf1^ is a cyclin-dependent kinase inhibitor (CKI). HDACi induces p21 expression by re-activating hyperacetylation of H3 and H4 in its promoter region (Richon et al., 2000). Furthermore, depsipeptide induces p53 phosphorylation at Thr 18, which is a requirement for subsequent p53 acetylation at K373/382 and p21 activation (Wang et al., 2012). In liver cancer, the HDAC8-selective inhibitor PCI-34051 can induce p21 expression and G2-M phase cell cycle arrest (Tian et al., 2015). Nevertheless, p21 and p16 are activated by HDACi in a p53-independent manner (Yoshida and Horinouchi, 1999). Namely, SIRT7 indirectly modulates p21-mediated cell cycle arrest by elevating p53 activity. SIRT7 physically binds and deacetylates P300/CBP-associated factor (PCAF) at K720, and this interaction is enhanced under conditions of glucose deprivation. As a result, PCAF binding to MDM2 is promoted, resulting in a triggering of MDM2 degradation via the ubiquitin-dependent proteasome pathway (Lu Y. F. et al., 2020). Finally, in p21-KO lymphomas, p27^*Kip1*^ (*CDKN1B*) functions in a p21-independent manner to induce cell cycle arrest after SAHA treatment (Newbold et al., 2014).

### Apoptosis

Apoptosis is a physiologically programmed cell death pathway that is essential for the maintenance of organismal homeostasis. Apoptosis is controlled by the B-cell lymphoma 2 (Bcl-2) family of proteins, which includes both pro-survival and pro-apoptotic proteins that control cell fate (Singh et al., 2019).

Regarding the intrinsic apoptotic pathway, the Bcl-2 interacting mediator of cell death (Bim), a Bcl-2 homology 3 (BH3)-only proapoptotic protein, is upregulated by depsipeptide via FoxO1 acetylation (Yang et al., 2009). Panobinostat elevates Sry-box transcription factor 7 (SOX7) expression and suppresses lung cancer cell proliferation. Mechanically, SOX7 triggers apoptosis by preventing Bim from proteasome-mediated degradation (Sun et al., 2019). The N-terminal truncated form of p63, ΔNp63, belongs to the p53 family, but acts as an oncoprotein. In squamous cell carcinoma, HDAC1 and HDAC2 form a complex with ΔNp63 to suppress the proapoptotic gene expression such as *p53 upregulated modulator of apoptosis (PUMA)* (Ramsey et al., 2011).

Histone deacetylase inhibitors treatment also affects the anti-apoptotic members of the Bcl-2 family. Specifically, depsipeptide induces apoptosis by decreasing the expression of pro-survival factors Bcl-2 and B-cell lymphoma-extra-large (Bcl-xL) (Adams and Eischen, 2016; Adams et al., 2016). The HDAC6-selective inhibitor ricolinostat exerts pronounced anti-lymphoma effects both alone and in combination with the alkylating agent bendamustine, by impairing the activation of caspase 8, -9, -3, and the Bcl-2 family (Cosenza et al., 2017). Myeloid cell leukaemia 1 (Mcl-1) is an E3-bound, anti-apoptotic protein that is involved in mitotic arrest (Senft et al., 2018). HDACi-induced Mcl-1 phosphorylation likely promotes apoptosis, whereas mutant phosphorylated Mcl-1 resists HDACi by binding to BH3-only proapoptotic proteins (Tong et al., 2018).

p53 is a crucial activator of apoptosis. HDAC1-3 all downregulate p53 activity, which represses p53-mediated activation of the pro-apoptotic gene BAX (Juan et al., 2000). Acetylation of p53 at K120 upregulates apoptotic peptidase activating factor 1 (Apaf-1) in the mitochondria (Yun et al., 2016). Under genotoxic stress, HDAC5 deacetylates p53 at K120, which activates pro-apoptotic target genes (Sen et al., 2013). Furthermore, HDAC6 directly deacetylates p53 at K120, which is required for p53-induced apoptosis in tumors with AT-rich interaction domain 1A (ARID1A) mutations (Bitler et al., 2017).

In summary, HDACi promote apoptosis via the intrinsic mitochondrial pathway, decreasing the expression of key anti-apoptotic factors (eg. Mcl-1, Bcl-2, and Bcl-xL), and/or increasing the expression of pro-apoptotic proteins (eg. BAX, Bim, Noxa and PUMA). HDACi also facilitate the activation of extrinsic apoptotic pathways, such as TRAIL (Nebbioso et al., 2017), driving mitochondrial outer membrane polarization (MOMP) and ultimately caspase-mediated cell death.

### Degradation System

The modulation of protein degradation is of critical importance for cell function. Protein degradation occurs via two major pathways: the ubiquitin-dependent proteasome pathway and autophagy system.

#### Autophagy

Autophagy is a degradation process whereby autophagosomes engulf and recycle nutrient sources in response to energetic demands and organelle turnover (Mizushima et al., 2008; Mizushima, 2018). Autophagy can be effectively promoted by HDACs. For example, depletion of HDAC10 perturbs autophagy flux through increased LC3-II/I, and the accumulation of p62 and acidic vesicular organelles. HDAC10 inhibition results in increased sensitivity to cytotoxic reagents (Oehme et al., 2013). Sirt1 also forms complexes with autophagy related protein 5 (ATG5), ATG7 and ATG8 to promote autophagy, with organelles in Sirt1^–/–^ mice being markedly damaged (Lee et al., 2008). The SIRT1 and -2 inhibitor tenovin-6 activates p53 and seems to be a specific regulator of mitochondrial acetylation (Lain et al., 2008; Scholz et al., 2015). Tenovin-6 suppresses Ewing sarcoma cells by regulating the NOTCH signaling pathway (Ban et al., 2014), and perturbs autophagic flux in chronic lymphocytic leukemia (CLL) cells and pediatric soft tissue sarcoma cells (Yuan et al., 2017). However, HDACs also interrupt autophagy, and various HDACi induce cancer cell death by promoting autophagy. In HDAC10-KO HeLa cells, chaperone-mediated autophagy (CMA) instead of macroautophagy is activated by the accumulation of lysosome-associated protein type 2A (LAMP2A)-positive lysosomes and the degradation of CMA substrate glyceraldehyde-3-phosphate dehydrogenase (GAPDH) (Obayashi et al., 2020). In response to serum starvation or oxidative stress, SIRT2 inhibition induces acetylated FoxO1 to locate in the cytoplasm, accelerating autophagy through interaction with ATG7 (Zhao et al., 2010). Under nutrient-rich conditions, FoxK1/2 bind to HDAC complex and restricts autophagic flux through the transcriptional repression of autophagy gene (Bowman et al., 2014). Under condition of nutrient deprivation, inhibition of the AKT serine/threonine kinase pathway facilitates nuclear import of FoxO3, which competitively replaces FoxK to bind the autophagy-associated gene promoters and upregulation of autophagy (Brunet et al., 1999; Bowman et al., 2014). Moreover, VPA activates autophagy by blocking HDAC1-mediated regulation of AKT pathway (Sun et al., 2020). The nutrient-sensor mammalian target of rapamycin (mTOR) negatively modulates downstream Unc-51-like autophagy activating kinase 1 (ULK1) that is involved in the non-transcriptional autophagic pathway. SAHA induces mTOR suppression, which ultimately activates autophagy by upregulating ULK1 (Gammoh et al., 2012). Of note, SAHA-induced autophagy seems to serve as a pro-survival mechanism to ameliorate SAHA-induced apoptosis by downregulating apoptotic factors (Gammoh et al., 2012). VPA also induces Sae2 [C-terminal-binding protein interacting protein (CtIP) in human] degradation in an autophagy related manner to impair HR-mediated DNA repair (Robert et al., 2011). As such, it seems that autophagy performs a dual role in DNA damage repair, depending on the cell states or DNA damage degree (Guo and Zhao, 2020).

#### Proteasome-Dependent Degradation

In addition to autophagy, proteasome-dependent degradation is also critical for cell function. HDACs target various E3s to affect basal cellular function. For example, panobinostat upregulates the E3 cIAP2 that causes the ubiquitination and proteasomal degradation of MRE11, elevating cellular sensitivity to chemoradiation (Nicholson et al., 2017). HDAC6 also modulates aggresome formation and the clearance of polyubiquitinated and misfolded proteins (Kawaguchi et al., 2003). In terms of therapeutic development, suppressing the aggresome pathway results in the accumulation of misfolded proteins, causing autophagy-associated DNA damage and apoptosis of cancer cells (Rodriguez-Gonzalez et al., 2008). Proteasome inhibitors (PIs), such as the United States Food and Drug Administration (FDA)-approved bortezomib (BTZ), have similar roles in preventing the degradation of polyubiquitin-misfolded proteins, which increases the production of ROS and disturbs DNA repair in tumor cells (Perez-Galan et al., 2006). However, long-term treatment with BTZ leads to drug-resistance in most patients. Low concentrations of HDACi combined with BTZ can downregulate anti-apoptotic proteins and upregulate pro-apoptotic proteins, thus accelerating cell death (Dai et al., 2008; Wang J. et al., 2019). Combining the HDAC6-selective inhibitor tubacin with BTZ induces significant anti-tumor activity triggering c-Jun NH2-terminal kinase (JNK)-caspase signaling and endoplasmic reticulum (ER) stress (Hideshima et al., 2005; Nawrocki et al., 2006). Another HDAC6 inhibitor, WT161, promotes the accumulation of acetylated tubulin and overcomes BTZ resistance to promote multiple myeloma (MM) cell death (Hideshima et al., 2016). RTS-V5, a dual inhibitor that targets HDAC6 and the 20S subunit of the proteasome, also possesses potent and selective anti-tumor activity in leukemia and MM cell lines (Bhatia et al., 2018).

### Epithelial-Mesenchymal Transition, Cancer Stem Cells, and Senescence

Epithelial-mesenchymal transition (EMT) is characterized by the loss of the tight intercellular connections normally found in epithelial cells that then undergo cytoskeleton rearrangement and adopt the mesenchymal cell phenotype, which is associated with migration. Notably, cancer cell migration and invasion are promoted by a series of EMT-associated factors (such as SNAIL, ZEB, SLUG and TWIST) (Kim K. K. et al., 2018). These factors induce EMT-related stem cell properties and promote tumorigenesis via PTMs (Mani et al., 2008; Tam and Weinberg, 2013; Ye et al., 2015; Dai et al., 2020). S-nitrosylation of HDAC2 is regulated by endothelial nitric oxide synthase (eNOS) that is a crucial enzyme for NO synthesis, allowing ZEB1 re-activation (Cencioni et al., 2018). During hypoxia, HDAC3 is essential for the activation of mesenchymal gene expression by the interaction with WD repeat domain 5 (WDR5) (Wu et al., 2011). The transforming growth factor-β (TGF-β)-SMAD signaling pathway is the most important EMT stimulation pathway. HDAC6 also has an essential role in EMT by activating SMAD3 (Shan et al., 2008). SMAD3 and -4 induce *SIRT7* transcriptional repression by forming a complex with HDAC8. HDAC8 inhibition significantly suppresses TGF-β signaling via SMAD-SIRT7 axis, and as a consequence, attenuates lung metastases of breast cancer (Tang et al., 2020). Class I HDACi 4SC-202 notably attenuates TGF-β-induced EMT (Mishra et al., 2017). By contrast, HDAC10 exhibits a potential TSG role by downregulating Sry-box transcription factor 9 (SOX9) in KRAS-driven lung adenocarcinoma. Furthermore, HDAC10 deficiency results in TGF-β pathway activation, leading to the induction of SOX9 and KRAS-expressing stem-like tumor growth (Li et al., 2020). Cancer-associated fibroblasts (CAFs) secrete extracellular matrix (ECM) that assists tumor progression and invasion. Scriptaid, a selective inhibitor of HDAC1, −3, and −8, represses TGF-β-mediated CAF by inhibiting ECM secretion and cell invasion (Kim D. J. et al., 2018). Of note, E-cadherin inhibits EMT, thus reduced E-cadherin expression indicates that “stemness” is increasing in cancer cells. HDAC and the 3-hydroxy-3-methylglutaryl coenzyme A reductase (HMGR) dual inhibitor JMF3086 restores E-cadherin expression and attenuates vimentin expression and stemness in NSCLC, which recovers sensitivity to gefitinib which is an epidermal growth factor receptor (EFGR) tyrosine kinase inhibitor (TKI) (Weng et al., 2019).

Cancer stem cells are hard to eradicate and prone to drug-resistance. HDAC3 interacts with p53 and forms complexes with tumor antigens melanoma antigen family A2 (MAGE-A2), establishing the resistance of melanoma cells to chemotherapeutic agents (Monte et al., 2006). In refractory and recurrent leukemia, HDAC8-selective inhibitor significantly restores acetylation and p53 activity, inducing apoptosis of AML cells but not of normal hematopoietic stem cells (Qi et al., 2015). In addition, SIRT1 inhibition increases the efficiency of BCR-ABL TKI imatinib mesylate to eliminate quiescent leukemia stem cells by reactivating p53 (Li et al., 2012).

**FIGURE 4:**
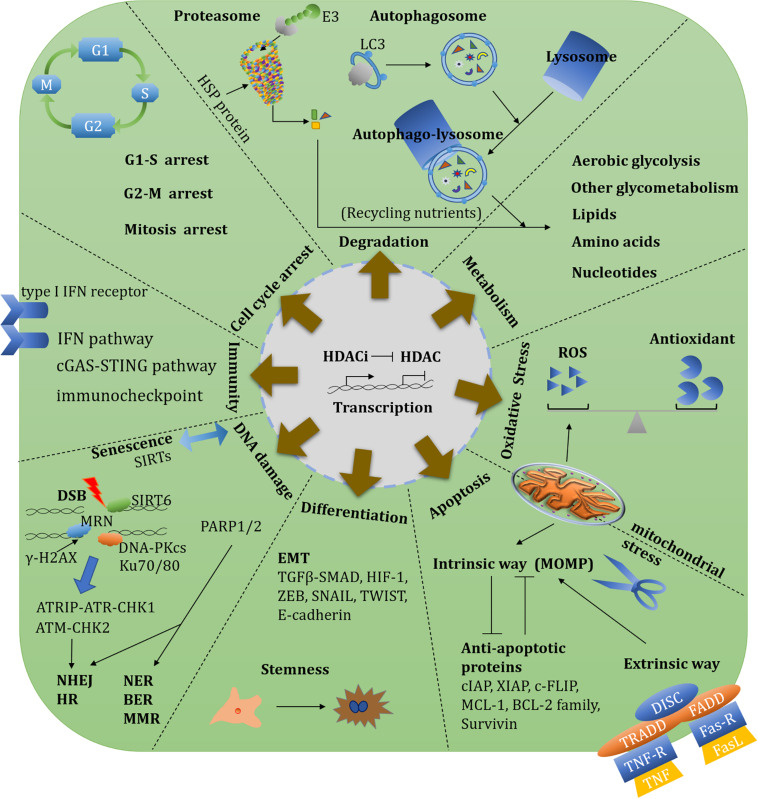
Overview of HDAC-involved biological functions and therapeutic targets. An overview of HDAC-involved biological functions including transcription, metabolism, oxidative stress, redox, protein degradation, cell cycle, DNA damage repair, apoptosis, angiogenesis, EMT, immunity, and stemness. There diverse functions could establish single or synergistic therapeutic targets.

Sirtuins are also closely involved in aging-related oncogene expression. Both SIRT6 and SIRT7 modulate long interspersed elements-1 (LINE-1, L1) expression and retrotransposition. SIRT6 mono-ADP-ribosylates the Krüppel-associated box domain-associated protein 1 (KAP1/TRIM28) and facilitates the KAP1 interaction with HP1α, resulting in the packaging of L1 elements into heterochromatin. SIRT7 directly binds L1 elements and promotes L1 sequences association with the nuclear lamina protein (Lamin A/C) by deacetylating H3K18 (Van Meter et al., 2014; Vazquez et al., 2019). These repressive functions of L1 highlight the protective roles of SIRTs on genome stability through preventing retrotransposition events. SIRT6 and SIRT7 deficiency result in aberrant heterochromatin and L1 activation especially in age-related diseases. Nucleoside reverse-transcriptase inhibitors can reverse a SIRT6 and SIRT7 deficiency by upregulating different immune signalings, such as the type I IFN pathway and cyclic GMP-AMP synthase (cGAS)-stimulator of interferon genes (STING) pathway, respectively (Simon et al., 2019; Bi et al., 2020; Figure 4).

## HDAC Inhibitors in Cancer Therapy

In the 1970s, sodium butyrate was discovered to transform red leukemia cells into normal cells, and to resynthesize hemoglobin. This process was accompanied by strong histone hyperacetylation, and resulted in the discovery of the first HDACi (Ginsburg et al., 1973; Riggs et al., 1977; Vidali et al., 1978). In Tsuji et al. (1976) isolated the first natural HDACi, TSA, which was derived from *Streptomyces hygroscopicus*. Following the discovery of TSA, trapoxin was isolated from fungi and also found to act as an HDACi (Itazaki et al., 1990). A number of natural inhibitors have since been extracted from fungi, marine life, and plants that contain sulfur, polyphenol, flavonoid, terpenoid, selenium, and other organic molecules (Newkirk et al., 2009; Lascano et al., 2018; Singh et al., 2018). At present, HDACi are mainly divided into four categories following the FDA approval: (i) hydroxamic acids or hydroxamates, such as SAHA, panobinostat and belinostat; (ii) cyclic peptides, including depsipeptide; (iii) benzamides, such as chidamide; and (iv) short-chain fatty acids, including VPA (Li and Zhu, 2014; Seto and Yoshida, 2014; Li and Seto, 2016; Table 3).

**TABLE 3:** Current clinical trials involving the use of HDAC inhibitors to treat cancer.

Chemical class	Drug name (synonyms)	HDACs	Current status
Hydroxamic acids	Vorinostat (SAHA)	Class I, II, and IV	FDA (2006)
	Belinostat (PXD101; PX105684)	Class I, II, and IV	FDA (2014)
	Panobinostat (LBH589)	Class I, II, and IV	FDA and EMA (2015)
	Resminostat (RAS2410; 4SC-201)	Selective- HDAC1, -3, -6	Phase II
	Givinostat (ITF2357)	Selective-HDAC1, -3	Phase II
	Pracinostat (SB939)	Classes I, II, and IV but except HDAC6	Phase II/III
	Abexinostat (CRA 024781; PCI-24781)	Class I and HDAC10	Phase I/II/III
	Quisinostat (JNJ-26481585)	Class I, II, and IV	Phase I/II
	MPT0E028	HDAC -1, -2, -6	Phase I
	Nanatinostat (CHR-3996)	HDAC1-3	Phase I
	CUDC 101	Class I and II HDAC, EGFR and HER2	Phase I
	Fimepinostat (CUDC-907)	HDAC1, -2, -3, -6, -10 and class I PI3K	Phase I
Benzamides	Chidamide (Tucidinostat; HBI-8000; Epidaza)	HDAC1, -2, -3, -10	Chinese FDA (2015)
	Entinostat (MS-275)	HDAC1-3	Phase II/III
	Rocilinostat/Ricolinostat (ACY1215)	Selective-HDAC6	Phase I/II
	Tacedinaline (N-acetyldinaline; CI-994)	HDAC1-3	Phase II/III
	Mocetinostat (MGCD0103)	HDAC1, -2, -3, -11	Phase I/II
	Domatinostat (4SC-202)	HDAC1, -2, -3, -5, 9, -10, -11 and LSD1	Phase I/II
Cyclic peptides	Romidepsin (FK 228; FR 901228; NSC 630176)	HDAC1, -2, -4	FDA (2009)
Fatty acids	Valproic acid	Class I, II	Phase I/II/III/IV
	AR-42 (OSU-HDAC42)	Class I and IIb	Phase I
	Pivanex (AN-9)	Class I and II	Phase II
	Sodium phenylbutyrate	HDAC and ER stress	Phase I/II
Sirtuins	Nicotinamide	SIRTs	Phase III
Others	CXD101	HDAC1-3	Phase I/II
	EDO-S101 (Tinostamustine)	Class I, IIb	Phase I/II
	Citarinostat (ACY241)	Class I, HDAC10	Phase I
	R306465	HDAC1, -8	Phase I

*FDA, Food and Drugs Administration; EMA, European Medicines Agency.*

### Efficiency of HDACi

From preclinical studies to clinical trials, HDACi have demonstrated powerful therapeutic effects in various cancers. HDACi can significantly attenuate tumor burden by limiting tumor growth and restraining aberrantly proliferated vessels (Guerriero et al., 2017). HDACi can also induce DNA damage, cell cycle arrest, apoptosis and autophagy to promote cancer cell death mentioned above. Some novel SIRT inhibitors, such as MC2494, MHY2245, MHY2256, tenovin-6, and YC8-02, also perform diverse anti-tumor activities through mediating apoptosis or autophagy (Carafa et al., 2018, 2019; De et al., 2018; Tae et al., 2018, 2020; Li M. et al., 2019; Igase et al., 2020).

Activation of the immune response by HDACi could also be an effective innate method to prevent cancer relapse when administered in a regimen with immunotherapeutic (Guerriero et al., 2017; Wang X. et al., 2020). The class IIa HDACi TMP195 efficiently improves the durability of tumor reduction in breast cancer by strengthening the phagocytic role of macrophages that are involved in the IFNγ axis; it also activates the adaptive anti-tumor immune response. Upon immune checkpoint blockade, TMP195 combined with an anti-programmed cell death-1 (PD-1) regimen could significantly reduce the tumor volume and induce a durable response in breast cancer (Guerriero et al., 2017). Myeloid-derived suppressor cells (MDSCs) suppress T-cell functions and promote tumor metastasis via the formation of an immunosuppressive tumor microenvironment (Azzaoui et al., 2016; Betsch et al., 2018). Entinostat exhibits remarkable curative effects when combined with PD-1 and cytotoxic T-lymphocyte-associated antigen-4 (CTLA-4) blockade; it has been shown to significantly reduce the number of MDSCs (Kim et al., 2014). 5-azacytidine combined with entinostat can also suppress MDSCs by downregulating C-C chemokine receptor type 2 (CCR2) and C-X-C chemokine receptor type 2 (CXCR2) expressions that ultimately, stimulates MDSC differentiation into a macrophage-like phenotype (Lu Z. et al., 2020). In human epidermal growth-factor receptor 2-positive (HER2^+^) breast cancer, panobinostat combined with trastuzumab (anti-HER2) stimulates the release of CXCR3-reactive chemokines and enhances the recruitment of tumor-associated natural killer (NK) cells to achieve eradication of tumors (Medon et al., 2017).

The first FDA-approved HDAC inhibitor, SAHA, belongs to the hydroxamic acid class, approved to treat patients with cutaneous T cell lymphoma (CTCL). In many clinical trials, SAHA has proven effective against advanced and refractory tumors, alone or in combination with other inhibitors. Subsequently, the cyclic peptide romidepsin was approved by the FDA in 2009 to treat CTCL. Panobinostat and belinostat were both approved by the FDA in 2014 to treat peripheral T cell lymphoma (PTCL), with belinostat gaining additional approval from the European Medicines Agency (EMA). Both panobinostat and belinostat are classified as hydroxamic acids. Meanwhile, chidamide has become the first benzamide HDACi to be approved by the China Food and Drug Administration(CFDA) in 2015, for the treatment of relapsed and refractory PTCL^1^ ^,2^ ^,3^ ^,4^. Most SIRT inhibitors still remain in the preclinical stages. So far, only nicotinamide (vitamin B3) has been used to treat cancer in clinical trials (e.g., NCT02416739 and NCT00033436). Nicotinamide has shown a potential role in inhibiting non-melanoma skin cancers that are principally generated by UV (Chen et al., 2015). Compared to other HDACi, nicotinamide exhibits the most catalytic sites: it is predominantly sensitive to acetylation sites in nuclear proteins that are involved in diverse biological processes (Scholz et al., 2015).

### Application of Selective Inhibitors and Combination Therapy

With the development of HDACi, numerous clinical trials are ongoing or completed currently for cancer therapy. Many HDACi have already been approved for hematological malignancies and lymphomas, while clinical studies are ongoing for refractory, advanced and recurrent solid tumors (Table 4). For instance, the use of the HDAC6-selective inhibitor ACY1215 (also named rocilinostat or ricolinostat) as a regimen for relapsed or refractory lymphoma and MM is currently in phase I/II clinical trials [Lymphoma (NCT02091063), MM (NCT01323751, NCT01583283, NCT01997840)]. HDACi with multiple targets have also been developed and tested in clinical trials, such as the dual HDAC and phosphoinositide-3 kinase (PI3K) inhibitor CUDC-907 (also called fimepinostat), which has been reported to inhibit Myc transcriptional expression and reduce Myc-mediated proliferation of multiple cancer cell lines (Pei et al., 2016; Kotian et al., 2017; Pal et al., 2018; Guo et al., 2019). The safety, tolerability and efficacy of CUDC-907 has been assessed in phase I/II trials (Younes et al., 2016). CUDC-101 is another multiple-target inhibitor that blocks HDACs, the epidermal growth factor receptor (EGFR) and HER2 in head and neck squamous cell cancer (Galloway et al., 2015).

**TABLE 4:** Clinical trials investigating the single-agents or combined therapies in HDACi and other anti-neoplastic drugs.

HDACi	Synergetic drugs (targets)	Clinical trial phase	Cancer specificity	Clinical registration number
Vorinostat (SAHA, Zolinza)	−	Phase I/II (finished)	pediatric (3−18 years) relapsed solid tumor, lymphoma and leukemia; well-tolerant; basal safe dose recommendation (SDR) of 130 mg/m^2^/day with weekly dose escalation was determined Van Tilburg et al., 2019	NCT01422499
	Mogamulizumab (anti-CCR4 monoclonal antibody)	Phase I (finished)	CTCL; Mogamulizumab significantly prolongs progression-free survival compared with vorinostat Kim Y. H. et al., 2018	NCT00719875
	Hydroxychloroquine (autophagy inhibitor)	Phase I (finished)	Advanced renal and CRC; safety and preliminary efficacy; establishs maximum tolerated dose (MTD) HCQ and vorinostat Mahalingam et al., 2014	NCT01023737
Belinostat (PXD-101)	Cisplatin (Chemotherapy), Etoposide (Topoisomerase II inhibitor)	Phase I (finished)	Small Cell Lung Cancer and Neuroendocrine Cancers; 48?h infusion with cisplatin plus etoposide shows safety and activity Balasubramaniam et al., 2018	NCT00926640
	Warfarin (anticoagulation)	Phase I (finished)	Solid Tumors or Hematological Malignancies; cannot affect the pharmacokinetics and pharmacodynamics of warfarin Agarwal et al., 2016	NCT01317927
	Ibritumomab tiuxetan/Zevalin (anti-CD20 monoclonal antibody)	Phase II (finished)	Aggressive lymphomas; establishes clinical biomarkers but cannot achieve overall response rate (ORR) Puvvada et al., 2017	NCT01686165
	−	Phase II (finished)	PTCL and CTCL; monotherapy is well tolerated and efficacious Foss et al., 2015	NCT00274651
Entinostat (MS-275, NSC 706995)	Sargramostim (GM-CSF)	Phase II (finished)	Relapsed and refractory myeloid malignancies; well tolerated and efficacious clinical activity but lack of longer observation periods Norsworthy et al., 2016	NCT00462605
	Aldesleukin (interleukin-2, IL2)	Phase I/II	metastatic clear cell renal cell carcinoma (ccRCC); improves progression-free survival and overall survival Pili et al., 2017	NCT01038778
	Trastuzumab (anti-HER2+)	Phase I	HER2+ breast cancer; well tolerated and efficacious clinical activity Lim et al., 2019	NCT01434303
Panobinostat (LBH 589)	bortezomib, thalidomide (immunosuppresive and anti-angiogenic activity) and dexamethasone	Phase I / II	refractory or relapsed MM; well-tolerant; takes orally 20 mg as SDR with escalation schedule Popat et al., 2016	NCT02145715
	bortezomib and dexamethasone	Phase I / II	relapsed and refractory MM; improves patients’ outcomes; overall survival benefits with panobinostat over placebo with bortezomib and dexamethasone San-Miguel et al., 2016	NCT01023308
	Bicalutamide/Casodex (androgen receptor antagonist)	Phase I / II (finished)	Castration-resistant prostate cancer; well tolerated and increased radiographic progression-free survival Ferrari et al., 2019	NCT00878436
Chidamide (Tucidinostat, Epidaza)	Exemestane (steroidal aromatase inhibitor)	Phase III (finished)	Tucidinostat plus exemestane improves progression-free survival in patients with advanced, hormone receptor (+), HER2 (−) breast cancer that failed and progressed after previous endocrine therapy Jiang et al., 2019	NCT02482753
	−	Phase II (finished)	relapsed or refractory PTCL; exhibits significant single-agent activity and manageable toxicity Shi et al., 2015	ChiCTR-TNC-10000811
Fimepinostat (CUDC-907), (PI3K and HDAC Inhibitor)	−	Phase I (finished)	Performs good safety and tolerability; orally administers the schedule 5 days followed by a 2-day break (5/2) at 60 mg in refractory or relapsed lymphoma or MM Younes et al., 2016	NCT02674750
	Rituximab (murine-derived monoclonal antibody binds CD20), Venetoclax (Bcl-2 inhibitor), Bendamustine (alkylated DNA crosslinker)	Phase I	Relapsed or refractory DLBCL; tolerable safety and durable anti-tumor activity particularly in MYC-driven patients Oki et al., 2017	NCT01742988
ACY-1215	Lenalidomide (immunomodulator) dexamethasone	Phase Ib	relapsed or refractory MM, safe, well-tolerated Vogl et al., 2017	NCT01583283
	bortezomib and dexamethasone	Phase I / II	relapsed or refractory MM, safe, well-tolerated, and active Vogl et al., 2017	NCT01323751

*“−” means monotherapy; Clinical trials mainly derived from United States National Library of Medicine.*

Combination drugs can inhibit tumorigenesis from different aspects. A clinical phase I trial of SAHA combined with the autophagy inhibitor MLN9708 shows potential for this regimen in advanced p53-mutant malignancies (NCT02042989). Hydroxychloroquine (HCQ) is a common autophagy-targeting reagent that has been used in clinical research (Mahalingam et al., 2014). Several phase I/II clinical studies (e.g., NCT02316340 and NCT01023737) have thus tested the safety, tolerability and pharmacological efficacy of SAHA combined with HCQ in solid tumors. A phase I/II trial targeting BTZ-resistant MM cancer has found that a synergistic regimen of using ricolinostat and dexamethasone could be safe and well-tolerated in affected patients (Vogl et al., 2017). A recent phase III clinical trial found that a combination of chidamide and exemestane has therapeutic potential for postmenopausal patients with advanced, hormone receptor-positive (HR^+^), HER2^–^ breast cancer and who have failed to respond to endocrine therapy (Jiang et al., 2019). Combining the SIRT1 inhibitor Ex527 with the WEE1 inhibitor MK-1775 produces efficient effects in lung cancers by impairing HR repair and mitotic catastrophe-associated apoptosis (Chen et al., 2017).

The overall survival (OS) and progression-free survival (PFS) are common and quite important indicators in the clinical trials. A phase I/II clinical study targets patients with metastatic clear cell renal cell carcinoma (ccRCC) by treatment with entinostat and high-dose interleukin-2 (IL2) that downregulates forkhead box P3 (Foxp3) expression and function of regulatory T cells (Treg). The median PFS reaches 13.8 months, and the median OS is 65.3 months (Pili et al., 2017) [NCT01038778]. A phase III trial enrolled 768 patients with relapsed MM exhibits that panobinostat shows the median OS of panobinostat (40.3 months) versus that of placebo (35.8 months) based on the existing treatments of bortezomib and dexamethasone. And patients who had received previous regimens such as immunomodulatory drug and bortezomib, median OS was only 25.5 months when received panobinostat, bortezomib, and dexamethasone versus that was merely 19.5 months who received placebo (San-Miguel et al., 2016) [NCT01023308]. These HDACi significantly exhibit potential median OS and PFS for patients with advanced tumors.

### Limitations of HDACi

However, in a randomized, double-blind and placebo-controlled phase III trial, vorinostat did not improve OS and could not be recommended as a therapy as a second-line or third-line drug for patients with advanced malignant pleural mesothelioma (Krug et al., 2015). Moreover, a phase III study that recruited 370 patients with Sézary syndrome or relapsed or refractory mycosis fungoides in CTCL from different countries of the world showed that the overall response rate to vorinostat was less efficient than that to mogamulizumab, a novel monoclonal antibody against CCR4 that significantly prolongs PFS (Kim Y. H. et al., 2018). The overall response to vorinostat in this study was significantly lower than reported in a previous study (Olsen et al., 2007). Besides, in a phase II trial, mocetinostat did not reverse chemoresistance in patients with previous gemcitabine-resistant leiomyosarcoma and could not significantly prolong the median PFS of patients (Choy et al., 2019).

Sirtuins constitute a relatively unique class of HDACs; as such, SIRT modulators are attracting great interest in the research community (Yang et al., 2017; Dai et al., 2018; Wang Y. et al., 2019). A novel SIRT7 inhibitor has been identified to inhibit tumor growth by blockade of the direct interaction of SIRT7 and p53 (Vakhrusheva et al., 2008; Kim J. H. et al., 2019). However, there are also several reports demonstrating an indirect interaction between SIRT7 and p53 (Barber et al., 2012; Lu Y. F. et al., 2020; Yu et al., 2020). Thus, it is urgent to develop specific SIRT inhibitors for cancer therapy according to reasonable mechanisms. It is worth noting that SIRT activators and sirtuin-activating compounds (STACs) have been developed and studied in clinical trials to investigate their anti-aging, anti-inflammatory and metabolic regulatory effects (Howitz et al., 2003; Hubbard et al., 2013; Sinclair and Guarente, 2014; Bonkowski and Sinclair, 2016; Carafa et al., 2016; Huang et al., 2018b). Nevertheless, the SIRT1 activator SRT2183 has also been shown to inhibit growth and to promote cell death by causing ER stress in glioma cells (Ye et al., 2019).

Besides, most of clinical trials of HDACi have reported many adverse effects, including bleeding caused by different grades of thrombocytopenia, susceptibility to infection caused by neutropenia, anemia caused by hemoglobin reduction, arrhythmia, myocardial hypertrophy, neurotoxicity, and gastrointestinal toxicity such as nausea, vomiting, fatigue, diarrhea as well as electrolyte disturbance such as hypophosphatemia and hyponatremia. The most adverse effect reported is cell death caused by continuous cytotoxicity (Medina et al., 1997; Zhu et al., 2001b), as these agents also kill immortalized and normal cells from different tissues (Lee et al., 1996; Burgess et al., 2004).

## Discussion and Perspectives

To date, only five HDACi have been approved by health authorities globally. Numerous clinical trials are currently evaluating the safety, application, and therapeutic benefits of HDAC inhibition when treating cancers, neurological disorders and other human diseases. Thus far, it is clear that FDA-approved HDACi have both beneficial and adverse effects. These effects might be accompanied by genomic instability, abnormal gene transcription, interference with chaperone protein function and free radical generation, which currently limit the therapeutic potential of HDACi.

Analyses of HDAC structural conformations and molecular mechanisms are necessary to improve treatment development. Complicating the application of HDACi is that the modification of the substrates by HDAC has spatio-temporal and tissue specificity. Consequently, dose-dependent and time-dependent treatments have different effects on gene expression regulation, various protein PTMs and chromatin remodeling. For now, combining different drugs to inhibit pathways such as tumor proliferation or angiogenesis, or to stimulate apoptosis requires more consideration.

Isolating the anti-cancer effects of HDACi and then synthesizing molecules with highly specific targets could be a promising avenue for cancer treatment in the future. Further investigation into combination treatments involving oncoprotein inhibitors and specific HDACi is also warranted. Overall, it seems that combination therapies have the advantage of reducing drug toxicity and lowering dose demand. SIRT protective factors should also be considered. Pending additional work to clarify HDACi that target specific HDACs or can be combined with other treatments, such as DNA methylation inhibitors or autophagy inhibitors, could be of great benefit to patients with cancers that have failed to respond to conventional treatments.

## Author Contributions

GL wrote the primary manuscript and YT revised the manuscript. W-GZ conceived and designed the manuscript. All authors contributed to the article and approved the submitted version.

## Conflict of Interest

The authors declare that the research was conducted in the absence of any commercial or financial relationships that could be construed as a potential conflict of interest.

## Footnotes

1 https://www.accessdata.fda.gov/scripts/cder/daf/

2 https://www.cancer.gov/about-cancer/treatment/drugs

3 https://www.ema.europa.eu/en

4 https://www.nmpa.gov.cn/

## Funidng

This work was supported by the National Key R&D Program of China (Grant No. 2017YFA0503900), Science and Technology Program of Guangdong Province in China (2017B030301016), Shenzhen Municipal Commission of Science and Technology Innovation (JCYJ20170818092450901), and Discipline Construction Funding of Shenzhen [(2016)1452].
